# Burden of Coronary Heart Disease and Cancer from Dietary Exposure to Inorganic Arsenic in Adults in China, 2016

**DOI:** 10.5334/aogh.3620

**Published:** 2022-04-28

**Authors:** Jialin Liu, Wenjing Song, Yiling Li, Yibaina Wang, Yuan Cui, Jiao Huang, Qi Wang, Sheng Wei

**Affiliations:** 1 MOE Key Lab of Environment and Health, Department of Epidemiology and Biostatistics, School of Public Health, Tongji Medical College, Huazhong University of Science and Technology, Wuhan, Hubei, 430030, PR China; 2 National Food Safety Risk Assessment Center, Key Laboratory of Food Safety Risk Assessment, Ministry of Health, Beijing, 10022, PR China; 3 Center for Evidence-Based and Translational Medicine, Zhongnan Hospital of Wuhan University, Wuhan, Hubei, 430030, PR China

## Abstract

**Background and Objectives:**

Inorganic arsenic (iAs) can cause a wide range of health problems, including coronary heart disease (CHD) and lung, bladder, and skin cancer. Although dietary iAs intake is the primary source of iAs, the burden of CHD and cancers from dietary iAs exposure in Chinese adults has not been well known.

**Methods:**

To estimate the iAs exposure level in Chinese adults’ diet, we systematically collected food-specific iAs concentrations in China from Chinese and English literature databases during 2000-2020. Food consumption was extracted from two nationwide food and nutrition surveys in China. The population attributable fraction was calculated based on the dose-response relationship between iAs and CHD risk. Combining the 2016 Chinese tumor registry data, we calculated the annual incidence of cancer from dietary iAs exposure to measure the disability-adjusted life year (DALY) in 2016.

**Findings:**

The total amount of daily foodborne iAs intake was 0.55 μg/kg bw/day among Chinese adults. The DALY of foodborne iAs-associated CHD was 3,017,510, which accounted for 10.18% of total CHD DALY in Chinese adults in 2016. Moreover, the carcinogenic DALY for lung cancer, bladder cancer, and skin cancer of Chinese residents in 2016 related to dietary iAs was 314.24, 9.89, and 167.32 thousand, accounting for 2.05%, 1.70%, and 35.5% of the total cancer burden, respectively.

**Conclusions:**

Our findings suggested that dietary iAs exposure causes a substantial disease burden in Chinese adults. More efforts for foodborne iAs control are critical to reducing the disease burden of CHD and cancer in China and other countries with similar dietary patterns.

## Introduction

Arsenic (As) is a metalloid that is widely distributed in the environment, in inorganic and organic forms via natural and anthropogenic activities. The potential routes of As accumulation in food supply include soil, contaminated water, and pesticide applications [1]. Diet has been considered an essential source of intake for general populations [2]. Grain food (e.g., rice/flour/coarse cereal) in the Asian diet constitutes a potential source of inorganic arsenic (iAs) intake for people. The health risks caused by dietary iAs intake have received increasing concern for decades [3,4].

Epidemiological studies have indicated that intake iAs has adverse effects on human cancer and non-cancer diseases. The iAs and its compounds were classified as a group I carcinogen by the International Agency for Research on Cancer [5]. Epidemiologic studies have shown the relationship between As exposure level and lung, bladder, and skin cancer risk [6,7,8]. Besides its carcinogenicity, iAs also contributes to cardiovascular disease (CVD) risk [9,10]. The majority of early studies found a risk of disease outcomes only at high levels of iAs exposure in drinking water [11,12]. However, growing evidence has been reported regarding the association between water iAs exposure and the risk of coronary heart disease (CHD) at low-moderate levels (<100μg/L) [13,14]. As the concentration of iAs in drinking water decreases, the proportion of dietary iAs intake increases. Nevertheless, studies on dietary iAs-associated CHD risk are limited at present [15]. The contribution of foodborne iAs exposure to the burden of CHD and cancer is an essential issue of concern.

Nowadays, there is an increasing concern about the burden of disease caused by iAs. In 2007, the World Health Organization (WHO) launched an initiative to estimate the global foodborne burden of diseases [16]. The European Food Safety Authority Panel on Contaminants in the Food Chain has assessed foodborne As-related human health risks in European countries [17]. Rice is a primary food in the Chinese diet that has a high iAs content. CVD and cancer were the leading causes of the disease burden in 2016 [18]. However, to date, little is known about the burden of foodborne iAs-induced CHD and cancer in China. Thus, the CHD and cancer disease burden due to dietary iAs needs to be quantitatively assessed.

The present study estimated the nation-level concentration of iAs in daily food in Chinese by systematic literature search. Then, the dietary iAs exposure in Chinese adults was evaluated by combining the consumption data from the national level surveys and concentration of iAs in daily food. Furthermore, the burden of CHD and cancer attributable to food iAs was estimated by counterfactual analysis proposed by the WHO. This study estimated for the impact of iAs intake on the health of Chinese adults to improve public health policies and supervision in food contaminants in China and other regions or countries with similar dietary patterns.

## Methods

### Data source of food type-specific iAs concentrations

**Figure 1:**
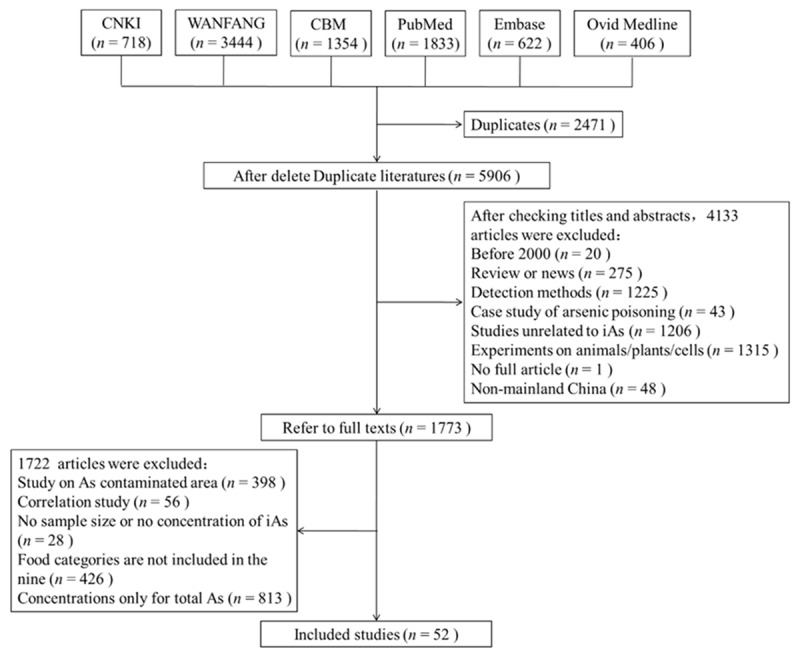
Flow chart of dietary inorganic arsenic (iAs) data retrieved from six databases in Chinese and English.

Data on iAs concentrations (mg/kg) in different food types were obtained by searching three Chinese databases (The General Library of Chinese Academic Journals, Wan-fang Data-Academic Journal Full Text library, and Chinese Biomedical Literature Database) and three English databases (PubMed, Embase, and Ovid MEDLINE) for relevant studies published from January 2000 to July 2019. The keywords for different database retrieval strategies are shown in Supplement Table S1. All literature reporting iAs concentrations in various food types in Mainland China was included. Details for literature search and exclusion are illustrated in ***Figure 1***. Sample-size weighted arithmetic means of iAs concentrations were calculated for each of the food types in studies using the following formula:


1 {C_k} = \;\mathop \sum \nolimits_{m = 1}^{m = n} ({N_{m,k}} \times {M_{m,k}})\,\,/\,\,\mathop \sum \nolimits_{m = 1}^{m = n} {N_{m,k}}

where *C_k_* means the weighted iAs concentration in the food *k* (mg/kg); *N_m_* means the sample size of food *k* in literature *m*; *M_m_* means the iAs concentration of the food *k* in the original study *m*; and *n* is the number of the articles searched of food *k*, as shown in Table S3.

### Data source of food consumption frequency

The specific food type consumption frequency (g/day) was obtained from two surveys (Supplement Table S2). One was the 5th China Total Diet Study conducted in 20 provinces from 2009 to 2013 [19]. The other was the 2015 China Household Survey Yearbook, which provided relevant data of 11 other provinces [20]. Each province, including two rural and one urban survey site, constituted a small market basket. A household food weighing method and 24-h dietary recall over three consecutive days were used in these two monitoring surveys to investigate the dietary patterns of Chinese adults. The consumption data collected from the population at the three survey sites in each province represent the average dietary mode of that province. Details for the two studies were mentioned before [21]. We categorized the food types into nine categories: grains (rice and other grains), potatoes, legumes/nuts, vegetables, fruits, meat, dairy products, eggs, and aquatic products.

### Estimated amount of daily foodborne iAs intake

The amount of daily foodborne iAs intake (DI, μg/day) and the estimated daily foodborne iAs intake by body weight (EDI, μg/kg body weight/day) were calculated by summing up the products of each type of food’s daily consumption frequency and the corresponding iAs concentration according to the following equations:


2 D{I_j} = \mathop \sum \limits_k ({C_k} \times {F_{kj}})

3 ED{I_j} = \mathop \sum \limits_k ({C_k} \times {F_{kj}})/B{W_j}

where *DI*_j_ (μg/day) and *EDI_j_* (μg/kg body weight/day) mean the total daily foodborne iAs intake of *j* province in China, *C_k_* means the iAs concentration in the food *k* (mg/kg), *F_kj_* means the daily consumption rate of the food *k* (g/day), and *BW_j_* means the average body weight (kg) in *j* province. The average body weight (*BW_j_*) of residents in regions of China was obtained from the China National Nutrition and Health Survey 2010 [22].

### Dose_response relationship between iAs exposure and CHD risk

To date, no related study reported the relationship between dietary iAs exposure and the risks of CHD in Chinese adults. Thus, we referred to a meta-analysis study of more than 0.4 million subjects worldwide from 10 studies to obtain the data [14]. This study regarded 10 μg/L of iAs in drinking water as the reference exposure. It calculated the risk of CHD at exposure to 10, 20, 50, 100, and 200 μg/L water iAs content assuming a constant log-linear association between log-transformed water iAs concentrations and relative risk of CHD (*P*-value for linear trend <0.05). Thus, iAs absorbed by the individual through food in this study was converted into a certain amount of water iAs content based on different bioavailability. Therefore, the incidence and mortality relative risk of CHD for 31 provinces can be obtained. The formulas were as follows:


4 {\rm C}_{\rm water} = ({\rm DI}_{\rm j} \times {{\rm Bio}_{\rm food}}){\rm{ /}}({{\rm Bio}_{\rm water}} \times {\rm M}_{\rm water})

5 {\rm{\;}}R{R_{incidence}} = 0.1826\,\, \times \,\,{\rm{ln(}}{C_{water}}{\rm{)}}\;\, + 0.568

6 {\rm{\;}}R{R_{mortality}} = 0.2992\,\, \times \,\,{\rm{ln}}({C_{water}})\;\, + \,\,0.278

where *C_water_* means the converted iAs concentration (μg/L) and *Bio_food_* and *Bio_water_* refer to the bioavailability of iAs through diet and drinking water, respectively [23]; *M_water_* refers to the daily intake of water for an adult in China (1.85 L/day) [24]; *RR_incidence_* and *RR_mortality_* mean the relative risk of foodborne iAs intake-induced CHD incidence and mortality.

### Estimation burden of CHD attributed to foodborne iAs intake

The disability-adjusted life year (DALY) is a metric used to evaluate the burden of a particular disease, estimated as the sum of corresponding years of lived with disability (YLD) and premature death-related years of life lost (YLL) caused by the disease [25]. A two-stage approach was adopted to calculate the DALY of CHD attributed to foodborne iAs exposure. First, the representative RR mentioned before was converted into the population attributable fraction (PAF) [26], defined as the percentage of outcomes reduced if exposure to a risk factor was reduced to the counterfactual level of minimum theoretical risk [27]. The conversion equation was as follows:


7 PA{F_{incidence}} = (R{R_{incidence}} - 1)/R{R_{incidence}}

8 PA{F_{mortality}} = (R{R_{mortality}} - 1)/R{R_{mortality}}

Then, the YLLs, YLDs, and DALY (in thousands) and age-standardized YLL, YLD, and DALY rates (per 100 000) of CHD attributed to foodborne iAs intake were calculated in the second stage. Liu et al. reported the all-cause CHD YLLs and YLDs in 2016 in all 33 province-level administrative units in China, which were available in this study [18]. We calculated the foodborne iAs exposure-related CHD burden according to the following equations:


9 A - YLL = PA{F_{mortality}} \times YLL{\__{all - cause}}

10 A - YLD = PA{F_{incidence}}\,\, \times \,\,YLD{\__{all - cause}}

11 A - DALY = A - YLL + A - YLD

12 PA{F_{total}} = {\rm{ }}A - DALY/DAL{Y_{ - all - cause}}

where *A-YLL, A-YLD*, and *A-DALY* mean the YLLs, YLDs, and DALY attributed to the foodborne iAs exposure. *PAF_mortality_* and *PAF_incidence_* mean the proportion of mortality and incidence of CHD due to foodborne iAs exposures, respectively. *PAF_total_* refers to the proportion of DALY related to foodborne iAs. The DALY rate was obtained by dividing the A-DALY by the number of adults in each province. Demographic data were obtained from the China Population and Employment Statistics Yearbook [28,29].

### Estimation of carcinogenic risk and cancer disease burden from dietary iAs exposure

The annual count (AC) (case/per year) is an indicator to evaluate the estimated carcinogenic risk of dietary exposure to iAs [30]. It is calculated as in equation (13).


13 AC = ({N_{pop}}*y*SF)/L{E_{pop}}

where, *N_pop_* is the total number of people exposed, y refers to the lifetime average daily iAs intake from dietary source, and *LE_pop_* is the life expectancy for exposed populations. *SF* refers to the slope factor, which is calculated as described below. Based on the Meta-regression coefficients of lung and bladder cancers caused by iAs exposure through the drinking water route, the SF (μg·kg^–1^·day^–1^)^–1^ values of lung and bladder cancers caused by dietary exposure to iAs in China were obtained by combining the cancer data and demographic characteristics of China [31,32].


14 {\rm SF} = {\rm IR}*({{\rm e}^{\beta}} - 1)*{\rm W}/{\rm Q}

In equation (14), IR is the cumulative lifetime incidence of cancer in the population, for which data were obtained from the 2016 Chinese tumor registry data [33]; β is the meta-regression coefficients of lung and bladder cancers; W is the average body weight of the Chinese population, with a value of 60 kg; Q is the average daily water consumption of the Chinese population. According to the US EPA’s IRIS database, the SF value for skin cancer is 0.0015 (μg·kg^–1^·day^–1^)^–1^.


15 DAL{Y_{ave/case}} = DAL{Y_{all - cause}}/Incidenc{e_{disease}}

16 DAL{Y_{iAs}} = AC*DAL{Y_{ave/case}}

where *DALY_all-cause_* consists of YLL and YLD, and its calculation formula has been reported in the previous literature [34]. The incidence and mortality cases of the three cancers in 2016 were extracted from the 2016 Chinese tumor registry data [33]. The DisMod Ⅱ software and WHO DALY calculation template were used to calculate the disease duration and *DALY_all-cause_* for each cancer [35]. Therefore, the average DALY per lung, bladder, and skin cancer case equals the total DALY divided by the number of cases (equation [15]). The disease burden of the three cancers due to dietary iAs exposure is calculated as shown in equation (16).

## Findings

We established a database including all retrieved eligible data of iAs concentrations in related food types (***Table 1*** and Supplement Table S3). The retrieval was independently completed by two individuals (Wenjing S and Jialin L), and the flow chart is shown in ***Figure 1***. The numbers of studies in various periods are shown in Supplement Table S4. A total of 52 studies with 45 296 unique iAs data points from 26 (municipalities or autonomous) provinces/cities were involved in our study. Grain was the most frequently reported food category, followed by aquatic products. The details of the results are shown in the Supplement Materials.

**Table 1:** Estimators of food type-specific inorganic arsenic concentrations in China.^a^


FOOD TYPES		NO. OF SAMPLES		MEAN (mg/kg)	
	
		NORTH	SOUTH	NORTH	SOUTH

Cereals	Rice	336	8501	0.094	0.085

	Other (except rice)	2041	1979	0.042	0.02

Legumes/Nuts		3009	3009	0.02	0.02

Potatoes		2880	2880	0.019	0.019

Meat		5105	5105	0.016	0.016

Eggs		4394	4394	0.013	0.013

Dairy products		3547	3547	0.011	0.011

Aquatic products		5035	5035	0.039	0.039

Vegetable		4533	4533	0.016	0.016

Fruit		3893	3893	0.011	0.011


^a^ Sample size-weighted estimators were calculated.

We pooled all data, and ***Table 1*** presents the iAs concentrations from nine food groups. The concentration of iAs in rice is similar in the northern and southern regions; however, the concentration of iAs in flour is slightly higher in the north. Vegetables and meat had the same mean iAs concentration (0.016 mg/kg), which was slightly higher than that of eggs (0.013 mg/kg). The lowest iAs concentrations were found in fruits and dairy products (0.011 mg/kg). The consumption of food in different provinces in China is shown in Supplement Table S5. As can be seen, that the adult rice consumption in the south and southwest provinces is higher than that in other regions. The vegetables are the second most consumed food group, following grains.

**Figure 2:**
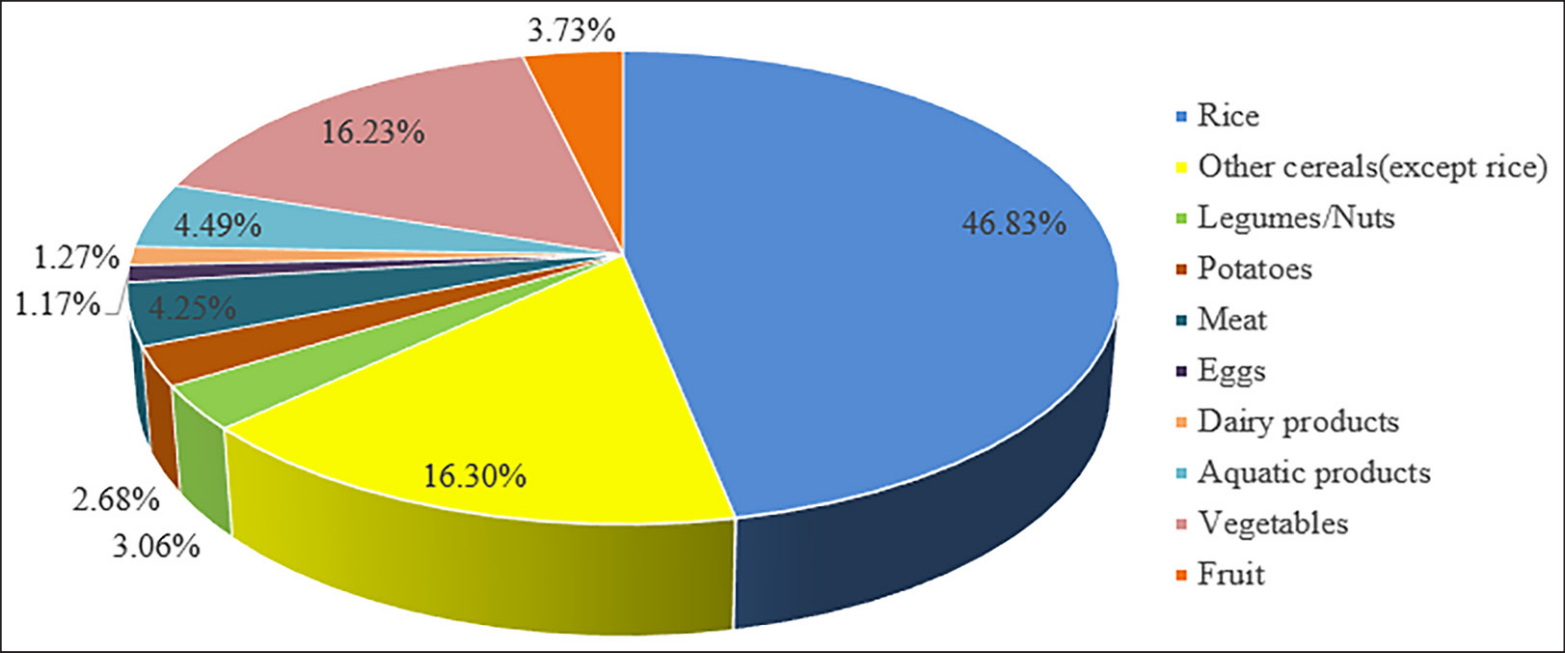
Proportion of inorganic arsenic contribution from different food groups in China.

The EDIs of iAs for residents in China and a specific province are shown in ***Table 2***. The total daily iAs intake was estimated to be 0.55 μg/kg bw/day (34.14 μg/day) among Chinese individuals, which was above the RfD of 0.3 μg/kg bw/day for iAs intake set by the USEPA (***Table 2***) [36]. The northeast region has the highest value of EDI compared with the other six regions of China. Jilin Province in the northeast region reported the highest EDI of iAs (0.94 μg/kg bw/day), about 2.2 times higher than Guangdong Province in the south of China. The primary source of foodborne iAs was rice, accounting for 46.83% of the total, followed by other grains, accounting for 16.30% of the total, and vegetables, accounting for 16.23% of the total (***Figure 2***).

**Table 2:** Average of estimated daily foodborne inorganic arsenic (iAs) intake in 31 provinces in China.


PROVINCE/CITY	FOODBORNE iAs INTAKE (μg/day)	FOODBORNE iAs INTAKE (μg/kg bw/day)^b^	PROVINCE/CITY	FOODBORNE iAs INTAKE (μg/day)	FOODBORNE iAs INTAKE (μg/kg bw/day)^b^

**China**	34.14	0.55			

**North**			Hubei	42.66	0.69

Beijing	41.03	0.60	Hunan	40.52	0.69

Hebei	35.57	0.54	**South**		

Inner Mongolia	35.32	0.53	Guangdong	24.51	0.42

Shanxi	24.71	0.38	Guangxi	39.53	0.70

Tianjin	30.43	0.45	Hainan	28.24	0.51

**Northeast**			**Southwest**		

Heilongjiang	37.24	0.57	Chongqing	34.31	0.59

Jilin	61.17	0.94	Guizhou	31.40	0.55

Liaoning	51.44	0.78	Sichuan	38.00	0.64

**East**			Tibet	28.34	0.50

Anhui	28.69	0.46	Yunnan	31.84	0.55

Fujian	44.48	0.75	**Northwest**		

Jiangsu	26.06	0.41	Gansu	35.13	0.56

Jiangxi	32.85	0.57	Ningxia	42.92	0.67

Shandong	29.09	0.44	Qinghai	33.80	0.53

Shanghai	40.25	0.63	Shaanxi	33.47	0.56

Zhejiang	54.81	0.90	Xinjiang	40.21	0.63

**Central**					

Henan	32.39	0.51			


^b^ The average body weight (bw) of residents in the regions of China was obtained from the China National Nutrition and Health Survey 2010.

**Figure 3:**
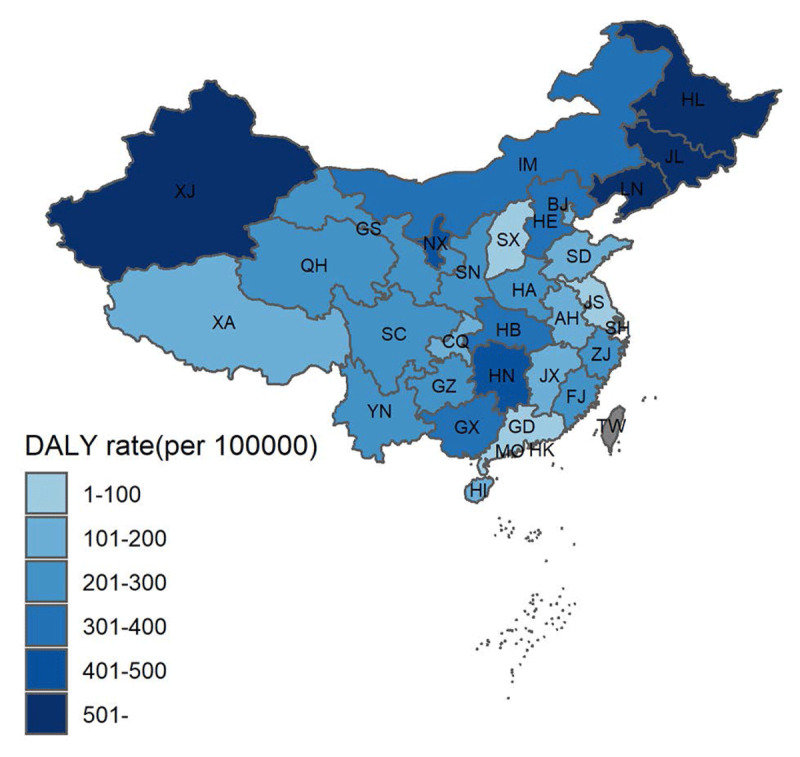
Map of age-standardized CHD DALY rate (per 100 000) induced by foodborne inorganic arsenic (iAs) intake in China.

The burdens of CHD attributed to foodborne iAs intake were estimated, and the results are shown in ***Table 3***, ***Figure 3***, and Supplement Table S6. The number of DALY attributed to foodborne iAs intake was 3,017,510 among Chinese, and the age-standardized DALY rate was 261.8 per 100,000 individuals (***Table 3***). Liaoning, Hunan, and Hebei were the top three provinces with the highest DALY numbers caused by foodborne iAs-related CHD. Qinghai, Hainan, and Tibet were the bottom three provinces with the lowest DALY number. After age-standardized, the DALY rate related to foodborne iAs intake in Jilin Province was more than 900 per 100 000 individuals. The DALY rate in Liaoning, Xinjiang, and Heilongjiang provinces was also more than 500 per 100,000 individuals. Notably, Guangdong Province had the lowest age-standardized DALY rate of only 27.47 per 100,000 individuals, about 33-fold lower than that of Jinlin Province. The age-standardized DALY rates of foodborne iAs-induced CHD were higher in the northern, northeast, northwest provinces, compared with the east, south coast, and southwest provinces of China, as shown in ***Figure 3***.

**Table 3:** Estimation of the burden of coronary heart disease attributed to foodborne inorganic arsenic (iAs) intake in 31 provinces in China in 2016.


PROVINCES	INCIDENCE-PAF	MORTALITY-PAF	YLLS (IN THOUSANDS) (95% UI)	AGE-STANDARDIZED YLL RATE (PER 100,000) (95% UI)	YLDS (IN THOUSANDS) (95% UI)	AGE-STANDARDIZED YLD RATE (PER 100,000) (95% UI)	DALY (IN THOUSANDS) (95% UI)	AGE-STANDARDIZED DALY RATE (PER 100,000) (95% UI)	PROPORTION OF DALY RELATED TO FOODBORNE iAs (%)

**China**	7.28%	10.30%	2931.8(2833.38–3025)	254.36(245.82–262.44)	85.72(59.21–117.07)	7.44(5.14–10.16)	3017.51(2892.6–3142.07)	261.8(250.96–272.6)	10.18%

**North**									

Beijing	10.08%	14.52%	53.7(46.19–61.77)	277.28(238.53–318.95)	2.39(1.66–3.28)	12.32(8.58–16.94)	56.08(47.86–65.05)	289.6(247.11–335.89)	14.25%

Hebei	7.92%	11.28%	233.51(204.47–261.51)	383.37(335.69–429.34)	5.31(3.64–7.29)	8.71(5.98–11.97)	238.82(208.11–268.8)	392.08(341.66–441.31)	11.17%

Inner Mongolia	7.81%	11.11%	85.62(75.11–97.9)	389.63(341.81–445.52)	2.02(1.4–2.77)	9.21(6.37–12.59)	87.64(76.51–100.67)	398.84(348.17–458.11)	11.00%

Shanxi	1.91%	1.78%	15.6(13.66–17.52)	50.05(43.84–56.23)	0.61(0.41–0.84)	1.94(1.32–2.69)	16.2(14.08–18.36)	52(45.16–58.92)	1.78%

Tianjin	5.44%	7.44%	26.43(22.74–30.13)	189.89(163.37–216.47)	0.86(0.6–1.19)	6.2(4.33–8.56)	27.29(23.34–31.32)	196.09(167.7–225.03)	7.36%

**Northeast**									

Heilongjiang	8.63%	12.35%	180.4(157.34–203.38)	527.92(460.46–595.17)	3.82(2.66–5.18)	11.17(7.79–15.15)	184.21(160.01–208.56)	539.09(468.24–610.32)	12.24%

Jilin	15.61%	22.44%	216.57(193.42–240.89)	907.2(810.21–1009.05)	4.18(2.89–5.78)	17.5(12.12–24.21)	220.75(196.31–246.67)	924.7(822.33–1033.26)	22.26%

Liaoning	13.30%	19.19%	261.17(226.98–294.41)	667.55(580.16–752.51)	7.81(5.44–10.75)	19.96(13.91–27.48)	268.98(232.42–305.16)	687.52(594.07–779.99)	18.95%

**East**									

Anhui	4.47%	5.91%	68.12(61.39–75.2)	133.31(120.14–147.18)	2.28(1.57–3.11)	4.46(3.07–6.08)	70.39(62.95–78.31)	137.77(123.21–153.26)	5.85%

Fujian	11.26%	16.25%	63.06(55.58–71.09)	199.22(175.6–224.59)	3.04(2.07–4.18)	9.6(6.53–13.21)	66.1(57.65–75.27)	208.81(182.12–237.8)	15.93%

Jiangsu	2.84%	3.29%	28.82(25.5–32.4)	41.77(36.96–46.96)	2.06(1.42–2.81)	2.99(2.06–4.08)	30.88(26.92–35.22)	44.75(39.02–51.04)	3.26%

Jiangxi	6.67%	9.36%	66.14(58.99–74.02)	182.82(163.08–204.63)	2(1.37–2.77)	5.54(3.8–7.65)	68.14(60.37–76.79)	188.37(166.88–212.27)	9.25%

Shandong	4.70%	6.27%	151.86(135.28–167.85)	183.81(163.73–203.16)	4.44(3.1–6.08)	5.37(3.75–7.36)	156.3(138.37–173.93)	189.18(167.48–210.52)	6.22%

Shanghai	9.80%	14.10%	29.91(25.92–34.04)	136.81(118.59–155.73)	2.42(1.67–3.33)	11.06(7.64–15.22)	32.32(27.59–37.37)	147.87(126.24–170.95)	13.65%

Zhejiang	14.16%	20.41%	96.81(84.84–110.56)	198.93(174.34–227.21)	6.17(4.21–8.49)	12.68(8.64–17.45)	102.98(89.05–119.06)	211.62(182.99–244.66)	19.89%

**Central**									

Henan	6.45%	9.02%	219.37(198.12–241.8)	290.54(262.4–320.24)	5.19(3.62–7.14)	6.87(4.79–9.46)	224.55(201.74–248.94)	297.41(267.19–329.7)	8.93%

Hubei	10.65%	15.36%	164.48(148.16–182.09)	330.68(297.86–366.09)	4.98(3.42–6.87)	10.01(6.89–13.81)	169.46(151.58–188.96)	340.69(304.74–379.9)	15.16%

Hunan	9.89%	14.24%	241.62(216.75–275.06)	432.98(388.42–492.91)	5.76(3.96–7.86)	10.32(7.09–14.09)	247.38(220.71–282.92)	443.3(395.51–506.99)	14.10%

**South**									

Guangdong	1.77%	1.54%	23.72(21.06–26.41)	25.9(23–28.84)	1.43(0.98–1.97)	1.56(1.07–2.15)	25.15(22.05–28.38)	27.47(24.08–30.99)	1.55%

Guangxi	9.53%	13.70%	139.3(123.66–156.35)	366.1(325.01–410.91)	3.4(2.32–4.69)	8.93(6.1–12.34)	142.7(125.99–161.04)	375.03(331.12–423.24)	13.56%

Hainan	4.21%	5.49%	9.12(8.02–10.37)	123.95(109.02–140.98)	0.28(0.19–0.38)	3.8(2.62–5.17)	9.4(8.22–10.76)	127.74(111.64–146.15)	5.44%

**Southwest**									

Chongqing	7.36%	10.42%	48.04(42.31–54.43)	186.42(164.19–211.2)	1.84(1.25–2.53)	7.12(4.86–9.81)	49.88(43.57–56.96)	193.54(169.05–221.01)	10.26%

Guizhou	5.95%	8.24%	56.18(48.42–64.77)	203.3(175.23–234.42)	1.49(1.02–2.05)	5.4(3.68–7.4)	57.67(49.44–66.82)	208.7(178.91–241.82)	8.16%

Sichuan	8.93%	12.81%	175.95(154.04–200.29)	253.41(221.86–288.47)	6.13(4.18–8.37)	8.83(6.02–12.05)	182.08(158.22–208.66)	262.24(227.88–300.52)	12.62%

Tibet	4.27%	5.59%	3.46(2.98–4.09)	137.61(118.64–162.63)	0.06(0.04–0.09)	2.48(1.66–3.43)	3.52(3.02–4.18)	140.09(120.3–166.06)	5.56%

Yunnan	6.17%	8.59%	76.04(67.54–86.02)	198.05(175.93–224.05)	1.76(1.19–2.46)	4.58(3.11–6.4)	77.79(68.74–88.47)	202.63(179.05–230.45)	8.51%

**Northwest**									

Gansu	7.73%	10.99%	59.37(52.88–66.74)	273.94(243.99–307.96)	1.49(1.02–2.04)	6.88(4.73–9.41)	60.86(53.9–68.78)	280.83(248.71–317.37)	10.88%

Ningxia	10.74%	15.49%	24.35(21.21–27.66)	446.23(388.67–506.74)	0.56(0.39–0.78)	10.27(7.06–14.27)	24.91(21.6–28.43)	456.5(395.73–521.01)	15.34%

Qinghai	7.12%	10.06%	13.19(11.49–14.95)	277.07(241.42–314.14)	0.27(0.18–0.37)	5.66(3.88–7.81)	13.46(11.68–15.33)	282.73(245.29–321.95)	9.98%

Shaanxi	6.97%	9.82%	94.42(83.02–107.41)	290.76(255.66–330.76)	2.02(1.39–2.77)	6.21(4.29–8.53)	96.44(84.42–110.18)	296.96(259.95–339.29)	9.74%

Xinjiang	9.78%	14.07%	102.54(90.72–115.56)	551.91(488.29–621.98)	1.52(1.05–2.09)	8.18(5.63–11.25)	104.06(91.77–117.65)	560.09(493.92–633.23)	13.98%


Jilin Province had the highest PAF of incidence and mortality (0.156 and 0.224, respectively), approximately 9–15 times higher than the lowest PAFs (0.018 and 0.015, respectively) in Guangdong Province (***Table 3***). After evaluating the DALY rates and age-standardized YLL/YLD rates related to foodborne iAs intake, the average proportion of DALY related to foodborne iAs was 10.18% in China, 1.55% (Guangdong Province) to 22.26% (Jilin Province) among 31 provinces/cities.

**Figure 4:**
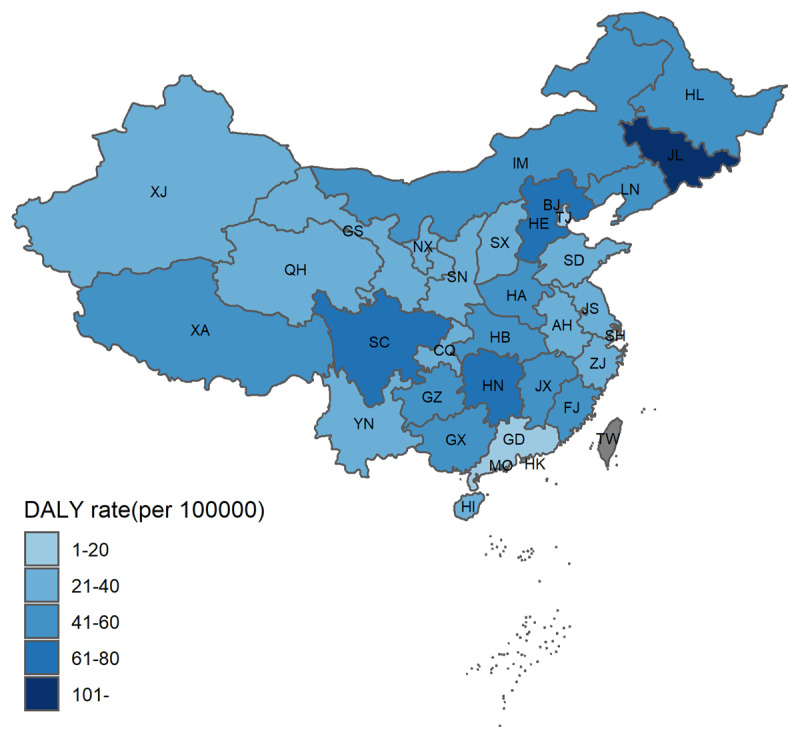
Map of age-standardized cancer DALY rate (per 100 000) induced by foodborne inorganic arsenic (iAs) intake in China.

The SF values for dietary iAs-induced lung and bladder cancer incidence were 0.001717 and 0.000139 (μg·kg^–1^·day^–1^)^–1^, respectively. The AC and DALY_ave/case_ for each cancer are shown in Supplement Table S7. In 2016, the carcinogenic DALY of Chinese residents exposed to iAs through diet was 491.46 thousand, accounting for 3.01% of three cancer DALY, of which the attributable DALYs for lung cancer, bladder cancer, and skin cancer was 314.24, 9.89, and 167.32 thousand, respectively (***Table 4***). In China, iAs exposure via dietary route will cause a loss of 35.54 years of healthy life for every 100 000 people. The DALY rate of cancers related to dietary iAs in Hebei Province, Inner Mongolia, Jilin Province, Liaoning Province, Hunan Province, and Sichuan Province is higher than the national average in China (***Figure 4***).

**Table 4:** DALY of lung, bladder and skin cancer attributed to foodborne inorganic arsenic (iAs) intake in 31 provinces in China in 2016.


PROVINCES	LUNG CANCER (IN THOUSANDS)	BLADDER CANCER (IN THOUSANDS)	SKIN CANCER (IN THOUSANDS)	TOTAL(IN THOUSANDS)	TOTAL PER 100,000	PROPORTION OF DALY RELATED TO FOODBORNE iAs (%)

**China**	314.24(297.81–326.57)	9.89(9.32–11.42)	167.32(143.13–180.11)	491.46(450.26–518.1)	35.54(32.56–37.47)	3.01%

**North**						

Beijing	3.09(2.53–3.66)	0.08(0.06–0.1)	0.81(0.53–1.18)	3.99(3.12–4.94)	18.37(14.37–22.71)	2.18%

Hebei	18.9(15.67–22.37)	0.69(0.53–0.93)	27.38(19.13–35.17)	46.97(35.33–58.47)	62.88(47.29–78.27)	5.72%

Inner Mongolia	5.62(4.73–6.64)	0.2(0.16–0.24)	7.11(5.31–8.72)	12.92(10.21–15.6)	51.29(40.5–61.89)	4.80%

Shanxi	6.21(4.94–7.56)	0.17(0.13–0.22)	5.99(4.53–7.45)	12.37(9.61–15.23)	33.58(26.09–41.37)	3.62%

Tianjin	2.31(1.95–3.86)	0.07(0.05–0.08)	0.62(0.48–0.88)	2.99(2.48–4.82)	19.16(15.86–30.84)	1.47%

**Northeast**						

Heilongjiang	14.36(11.94–17.03)	0.32(0.26–0.39)	5.15(4.15–6.78)	19.83(16.35–24.19)	52.21(43.03–63.68)	2.53%

Jilin	10.83(8.77–12.79)	0.35(0.27–0.42)	25.8(17.15–33.37)	36.98(26.2–46.58)	135.32(95.86–170.43)	9.99%

Liaoning	15.44(12.84–18.37)	0.44(0.33–0.53)	7.33(5.41–8.93)	23.21(18.58–27.83)	53.02(42.43–63.58)	2.74%

**East**						

Anhui	13.4(11.27–16)	0.49(0.41–0.6)	5.66(4.47–6.71)	19.55(16.14–23.31)	31.55(26.06–37.62)	2.68%

Fujian	13.07(10.78–15.8)	0.39(0.31–0.52)	5.03(3.97–6.54)	18.49(15.06–22.86)	47.73(38.87–59.01)	4.65%

Jiangsu	14.75(12.28–17.52)	0.47(0.38–0.57)	4.5(3.39–5.55)	19.72(16.06–23.64)	24.65(20.07–29.56)	1.84%

Jiangxi	12.58(10.71–14.81)	0.45(0.37–0.57)	7.8(6.33–9.3)	20.83(17.42–24.68)	45.37(37.93–53.75)	4.66%

Shandong	20.02(16.73–23.73)	0.61(0.51–0.75)	11.02(8.85–13.63)	31.66(26.1–38.11)	31.83(26.23–38.31)	2.06%

Shanghai	2.82(2.31–3.42)	0.09(0.06–0.11)	0.82(0.52–1.06)	3.73(2.9–4.59)	15.41(11.97–18.99)	1.67%

Zhejiang	15.81(12.99–18.97)	0.54(0.43–0.65)	4.13(3.04–5.39)	20.48(16.47–25.01)	36.63(29.46–44.75)	3.00%

**Central**						

Henan	21.89(18.3–25.83)	0.87(0.69–1.32)	17.17(13.99–20.78)	39.93(32.98–47.93)	41.89(34.6–50.28)	4.12%

Hubei	20.08(16.79–23.82)	0.61(0.5–0.73)	7.99(6.53–10.05)	28.67(23.81–34.61)	48.72(40.47–58.81)	3.59%

Hunan	24.85(20.83–30.04)	1(0.79–1.4)	24.45(18.5–29.98)	50.31(40.13–61.42)	73.74(58.82–90.03)	5.27%

**South**						

Guangdong	17.53(14.64–20.85)	0.49(0.41–0.62)	4.35(3.28–5.61)	22.37(18.33–27.09)	20.34(16.66–24.63)	2.36%

Guangxi	14.79(12.43–17.76)	0.51(0.4–0.82)	6.99(5.73–8.66)	22.3(18.56–27.24)	46.09(38.37–56.3)	5.03%

Hainan	1.65(1.32–2.06)	0.06(0.04–0.08)	0.7(0.52–1.03)	2.41(1.89–3.17)	26.26(20.63–34.6)	3.77%

**Southwest**						

Chongqing	7.14(5.71–8.9)	0.23(0.18–0.3)	2.85(2.23–3.83)	10.22(8.13–13.03)	33.52(26.66–42.75)	2.46%

Guizhou	6.3(5.18–7.53)	0.31(0.25–0.38)	9.54(7.09–12.03)	16.15(12.52–19.94)	45.42(35.23–56.1)	5.34%

Sichuan	31.13(25.2–36.57)	1.15(0.91–1.4)	21.68(17.49–25.99)	53.96(43.59–63.96)	65.31(52.77–77.41)	4.04%

Tibet	0.54(0.44–0.65)	0.04(0.03–0.07)	0.85(0.66–1.08)	1.43(1.14–1.79)	43.13(34.3–54.19)	26.75%

Yunnan	10.05(8.47–12.02)	0.38(0.32–0.48)	7.46(5.74–9.12)	17.89(14.52–21.62)	37.5(30.44–45.31)	4.75%

**Northwest**						

Gansu	5.03(4.19–6.01)	0.16(0.13–0.2)	2.27(1.88–2.66)	7.46(6.2–8.86)	28.58(23.77–33.96)	4.65%

Ningxia	1.69(1.37–2.06)	0.04(0.03–0.05)	0.34(0.28–0.41)	2.08(1.68–2.52)	30.76(24.92–37.37)	4.89%

Qinghai	1.24(1.01–1.48)	0.05(0.04–0.07)	0.72(0.59–0.86)	2.01(1.64–2.41)	33.9(27.58–40.63)	5.82%

Shaanxi	7.72(6.17–9.6)	0.29(0.23–0.4)	6.74(5.5–8.14)	14.75(11.9–18.14)	38.69(31.21–47.58)	4.77%

Xinjiang	3.86(3.19–4.57)	0.12(0.09–0.19)	1.3(1.04–1.56)	5.29(4.32–6.32)	22.05(18.02–26.34)	4.05%


## Discussion

This study estimated the national level of iAs exposure from the dietary route and its contribution to CHD and cancer-related DALY in Chinese adults. The average foodborne iAs intake was 34.14 μg/day and 0.55 μg/kg bw/day among the Chinese. Rice/flour/coarse cereal contributed the most significant proportion (63.13%) of dietary iAs intake, and there were significant regional differences in the intake of iAs. The foodborne iAs intake-related CHD burden was quantified with DALY number and age-standardized DALY rate, equaling 3,017,510 and 261.8 per 100,000 persons, respectively. Furthermore, the carcinogenic DALY for lung cancer, bladder cancer, and skin cancer in China attributed to dietary iAs was 314.24, 9.89, and 167.32 thousand, accounting for 2.05%, 1.70%, and 35.5% of the respective total cancer burden, respectively.

Dietary iAs exposure has been considered as an important issue, and there has been an increasing trend for dietary iAs intake in recent years. Previous studies have demonstrated that diet is the primary source of iAs exposure for people living in areas with a low iAs level in drinking water [2,37]. In The Fifth China Total Diet Study (in 2009–2013), the dietary iAs exposure in Chinese residents was 27.7 μg/d (range 10.8–44.6 μg/d), which was lower than the results in this study [19]. However, our result was similar to the previous exposure assessment in India, which indicated that the iAs exposure via rice of individuals in the three regions was 0.3 to 0.84 μg/kg bw/day [38]. Moreover, a dietary survey in Japan showed that iAs intake was 0.446 μg/kg bw/day [39]. It may be that the iAs concentration in rice has been increasing in recent years or that the bioavailability is not considered when assessing dietary iAs intake, which would overestimate the actual intake of the population [40].

Food is susceptible to contamination from natural and anthropogenic elements [41]. Grains, capable of accumulating iAs during growth, are the primary staple food among Chinese people, resulting in grains being a primary source for foodborne iAs intake. As rice/flour/coarse cereal contributed most to foodborne iAs intake, the limits of iAs contents in such food types may reduce iAs exposure among the Chinese. Meanwhile, the contribution of vegetables should not be ignored. Although the iAs concentration in vegetables was low, the iAs intake via vegetables ranked second for the high daily consumption. Our results demonstrated the critical role of rice/flour/coarse cereal consumption in foodborne iAs exposure, suggesting that less rice/flour/coarse cereal intake in the daily diet may effectively reduce the iAs intake.

Our study indicated that the burden of CHD attributable to dietary iAs is not negligible. The elevated risk of CHD in response to low, moderate, and high iAs in drinking water has been studied in a meta-analysis [14]. This review excluded cross-sectional and ecological studies to inform a more flexible dose_response relationship, addressing the shortcomings of previous studies that compared only the highest with the lowest iAs levels. Meanwhile, under the assumption of a log-linear relationship between iAs and the risk of CHD, we used DALY, which is an internationally accepted method of measuring disease burden created by the WHO, as an indicator to calculate the disease burden from dietary iAs [42]. A previous study reported that dietary iAs caused, on average, 669 DALY per 100,000 among CHD patients in 13 GEMS around the world, which indicated that dietary iAs intake caused significant but avoidable diseases of burden. Hence, a reduction in dietary iAs intake should be implemented to improve quality of life. In this study, the age-standardized DALY rate of dietary iAs intake-induced CHD was 261.8 per 100,000 in China, lower than 676 DALY per 100,000 for the disease burden of iAs-associated CHD in the Western Pacific (WPR B, including China) [43]. This may have resulted from the consumption or concentration of rice and other cereals, which was not separated from the global subregional attribution calculations.

In addition, our findings suggested that dietary iAs exposure causes a substantial cancer disease burden in Chinese adults. The iAs in food and drinking water is assumed to produce the same carcinogenic risk. Furthermore, the cancer disease burden from dietary iAs assessed in this study in China was similar to that calculated by the FERG working group [43]. In the assessment, Jilin, Hunan, and Sichuan provinces have the highest standardized cancer disease burden, which may be due to the high rice consumption. Although many studies on As have been based on drinking water intake, dietary intake of iAs is close to that of drinking water intake in areas with low iAs concentrations in water [44]. Therefore, although the complexity of measuring foodborne As exposure and the controversial linear dose response relationships for As-associated cancers make the assessment process uncertain, the disease burden caused by each As pathway need to be quantified.

Although our findings provided a nationwide estimation for foodborne iAs-associated CHD and cancer burden, several limitations should be mentioned in this study. First, some iAs concentrations in various food types were referred to published literature for journals, with a publication bias process. Moreover, the concentration of various foods at the provincial level could not be obtained due to missing data. Second, the health effect risk may be underestimated in this study. Dietary iAs intake was not accounted for some other food types (e.g., oil, sugar, pastry) due to unavailable data. Similarly, the iAs exposure from drinking water was not included in the present study as the amount of water consumed through the diet is difficult to quantify. In addition, the latest version of the disease burden data for CHD and cancer was not used as YLL and YLD data were lacking. Finally, in this study, the risk of disease from iAs in the diet and iAs in drinking water was the same to calculate the attributable disease burden. Therefore, more studies on dietary iAs and disease risk should be conducted in the future to reduce the uncertainty in the calculation of the attributable disease burden.

## Conclusions

Our study provided a nation-level estimation for the burden of disease by foodborne iAs in China. Our results indicated that dietary iAs exposure causes a significant disease burden, especially in areas with higher rice/flour/coarse cereal consumption. More attention to policies controlling dietary iAs intake is necessary for China and other regions or countries with similar dietary patterns.

## Additional File

The additional file for this article can be found as follows:

10.5334/aogh.3620.s1 Supplement Materials. The burden of coronary heart disease and cancer from dietary exposure to inorganic arsenic in adults in China, 2016.
