# Supplementary material for: Burden of Coronary Heart Disease and Cancer from Dietary Exposure to Inorganic Arsenic in Adults in China, 2016
Source: Ann Glob Health. 2022 Apr 28;88(1):28. doi: 10.5334/aogh.3620 (PMC9053568; doi:10.5334/aogh.3620)
Supplement: Supplement Materials. — The burden of coronary heart disease and cancer from dietary exposure to inorganic arsenic in adults in China, 2016. [file agh-88-1-3620-s1.pdf]

# Supplement Materials

## The burden of coronary heart disease and cancer from dietary exposure to inorganic arsenic in adults in China, 2016

Jialin Liu[1+], Wenjing Song[1+], Yiling Li[1], Yibaina Wang[2], Yuan Cui[1], Jiao Huang[3], Qi Wang[1*], Sheng Wei[1*]

[1]MOE Key Lab of Environment and Health, Department of Epidemiology and Biostatistics, School of Public Health, Tongji Medical College, Huazhong University of Science and Technology, Wuhan, Hubei, 430030, PR China.

[2]National Food Safety Risk Assessment Center, Key Laboratory of Food Safety Risk Assessment, Ministry of Health, Beijing, 10022, PR China.

[3]Center for Evidence-Based and Translational Medicine, Zhongnan Hospital of Wuhan University, Wuhan, Hubei, 430030, PR China.

[+] The authors contributed equally to this manuscript.

[*]Corresponding author: Prof. Sheng Wei and Prof. Qi Wang, School of Public Health, Tongji Medical College, Huazhong University of Science and Technology, Wuhan 430030, People's Republic of China. E-mail: shengwei@hust.edu.cn (S. W); wangqi_tj@hust.edu.cn (Q. W)

## Notes on the literature search for data on iAs concentrations in food

A total of 52 studies with 45 296 unique iAs data points were involved in our study. According to previous reports, cereals are the main source of dietary inorganic arsenic intake. Taking into account the differences in dietary habits between the north and the south, we separately calculated the concentration of grains and the intake of inorganic arsenic in rice. As shown in Table 1 and Supplement Table S3, due to limited sample size, iAs concentrations for other food groups could not be combined by province and only national averages were used.

**Table S1. Retrieval strategies and results about inorganic levels in Chinese food.**

| Databases | Retrieval strategy | Number of articles |
|---|---|---|
| CNKI # (In Chinese) | Title or Keyword: (arsenic or metal) * Title or Keyword: (food or diet) * Date: 2000-2019 | 718 |
| WANFANG # (In Chinese) | Title or Keyword: ( arsenic or metal) * Title or Keyword: ( food or diet) * Date: 2000-2019 | 3444 |
| CBMdisc # (In Chinese) | “arsenic and food” [Full Field] | 1354 |
| Pubmed | ((arsenic [Title/Abstract] AND food [Title/Abstract]) AND China [Title/Abstract]) AND ("2000/01/01"[PDAT] : "2019/07/24"[PDAT]) | 1833 |
| Embase | #1. (arsenic and (food or dietary or rice or fish or "sea food" or "aquatic procduct " or egg or wheat or flour or corn or milk or meat or shellfish or laver or kelp or vegetables or grain or friut or chicken or bean) and (china or chinese)).mp. [mp=title, abstract, heading word, drug trade name, original title, device manufacturer, drug manufacturer, device trade name, keyword, floating subheading word, candidate term word] #2. limit 1 to (abstracts and yr="2000 - 2019") | 622 |
| Ovid Medline | (TS=food or TS=dietary or TS=rice or TS=fish or TS="sea food" or TS="aquatic procduct " or TS=egg or TS=wheat or TS=flour or TS=corn or TS=milk or TS=meat or TS=shellfish or TS=laver or TS=kelp or TS=vegetables or TS=grain or TS=friut or TS=chicken or TS=bean) AND (ts=arsenic) AND (ts=china or ts= chinese) AND (Journal Article) AND yr="2000 - 2019" | 406 |

# China National Knowledge Internet (CNKI), WanFang and China Biology Medicine Disc (CBMdisc), all of them are the professional academic databases of China.

**Table S2. Provinces' information of China seven geographical regions in the 5th China Total Diet Study and the 2015 China Household Survey.**

| Survey | Provinces |
|---|---|
| The 5th China Total Diet Study (2009-2013) | **North:** Beijing, Hebei, Inner Mongolia<br>**Northeast:** Heilongjiang, Liaoning, Jilin<br>**East:** Shanghai, Jiangsu, Zhejiang, Jiangxi, Fujian<br>**Central:** Hubei, Hunan, Henan<br>**South:** Guangdong, Guangxi<br>**Southwest:** Sichuan<br>**Northwest:** Shaanxi, Ningxia, Qinghai |
| The 2015 China Household Survey Yearbook (2015) | **North:** Tianjin, Shanxi,<br>**East:** Anhui, Shandong<br>**South:** Hainan<br>**Southwest:** Chongqing, Guizhou, Yunnan, Tibet<br>**Northwest:** Gansu, Xinjiang |

**Table S3. Levels of inorganic arsenic (iAs) in various food types reported in Chinese literature from 2000 to 2019.**

| Food types | *n* | mean (mg/kg) | SD | sampling year | method of detection[a] | Study order[b] | Food types | *n* | mean (mg/kg) | SD | sampling year | method of detection[a] | Study order[b] |
|---|---|---|---|---|---|---|---|---|---|---|---|---|---|
| **1. Rice/Flour/Coarse cereal** | | | | | | | Livestock and poultry meat | 9 | 0.011 | 0.0072 | 2000 | 2 | [3] |
| Brown Rice | 1 | 0.21 | | 2007 | 1 | [12] | Livestock and poultry meat | 669 | 0.007 | | 2006-2007 | 2 | [10] |
| Brown Rice | 14 | 0.164 | 0.052 | 2008 | 1 | [15] | Livestock and poultry meat | 62 | 0.0091 | | 2010-2012 | 2 | [26] |
| Brown Rice | 446 | 0.208 | 0.047 | 2013 | 1 | [34] | Livestock meat | 66 | 0.019 | | 2010-2015 | 2 | [27] |
| Cereal | 1080 | 0.072 | | 2000 | 2 | [1] | Meats | 1080 | 0.028 | | 2000 | 2 | [1] |
| Cereal | 1800 | 0.013 | 0.006 | 2009 | 1 | [21] | Meats | 10 | 0.024 | | 2006 | 2 | [9] |
| Coarse cereals | 28 | 0.06 | | 2000 | 2 | [2] | Meats | 1800 | 0.004 | 0.002 | 2009 | 1 | [21] |
| Coarse cereals | 5 | 0.11 | 0.044 | 2000 | 2 | [3] | Mutton | 50 | 0.0075 | | 2005 | 2 | [7] |
| Coarse cereals | 61 | 0.019 | | 2010-2015 | 2 | [27] | Mutton | 50 | 0.0075 | | 2007-2008 | 2 | [13] |
| Flour | 33 | 0.026 | | 2000 | 2 | [2] | Pork | 50 | 0.0075 | | 2005 | 2 | [7] |
| Flour | 5 | 0.075 | 0.016 | 2000 | 2 | [3] | Pork | 50 | 0.0075 | | 2007-2008 | 2 | [13] |
| Flour | 54 | 0.0075 | | 2005 | 2 | [7] | Visceral meat | 64 | 0.132 | 0.259 | 2007 | 2 | [11] |
| Flour | 21 | 0.018 | | 2010-2015 | 2 | [27] | Visceral meat | 29 | 0.036 | 0.032 | 2007 | 2 | [11] |
| Grain | 10 | 0.058 | | 2006 | 2 | [9] | Visceral meat | 25 | 0.302 | 0.224 | 2007 | 2 | [11] |
| Grain | 923 | 0.018 | | 2006-2007 | 2 | [10] | Visceral meat | 50 | 0.026 | | 2007-2008 | 2 | [13] |
| Milled rice | 1653 | 0.0909 | 0.042 | 2012 | 4 | [32] | Visceral meat | 50 | 0.022 | | 2007-2008 | 2 | [13] |
| Polished rice | 21 | 0.082 | | 2010 | 1 | [24] | Visceral meat | 2 | 0.0437 | | 2009 | 2 | [18] |

| | | | | | | |
|---|---|---|---|---|---|---|
| Polished rice | 41 | 0.092 | | 2015 | 1 | [40] |
| Polished rice | 160 | 0.054 | | 2017 | 1 | [49] |
| Rice | 40 | 0.06 | | 2000 | 2 | [2] |
| Rice | 5 | 0.15 | 0.01 | 2000 | 2 | [3] |
| Rice | 50 | 0.077 | | 2005 | 2 | [7] |
| Rice | 38 | 0.161 | 0.035 | 2009 | 2 | [19] |
| Rice | 9 | 0.08 | | 2009 | 8 | [20] |
| Rice | 4 | 0.09 | | 2009 | 8 | [20] |
| Rice | 6 | 0.08 | | 2009 | 8 | [20] |
| Rice | 4 | 0.07 | | 2009 | 8 | [20] |
| Rice | 25 | 0.0504 | | 2010-2012 | 3 | [25] |
| Rice | 4188 | 0.072 | | 2010-2015 | 2 | [27] |
| Rice | 446 | 0.0584 | | 2011 | 1 | [28] |
| Rice | 54 | 0.0593 | | 2011 | 4 | [29] |
| Rice | 5 | 0.11 | 0.017 | 2011-2012 | 2 | [31] |
| Rice | 206 | 0.045 | 0.019 | 2013 | 1 | [33] |
| Rice | 446 | 0.109 | 0.024 | 2013 | 1 | [34] |
| Rice | 168 | 0.084 | 0.022 | 2014 | 2 | [37] |
| Rice | 300 | 0.1 | 0.02 | 2014 | 1 | [38] |
| Rice | 43 | 0.1118 | 0.0344 | 2015 | 1 | [41] |
| Rice | 260 | 0.0441 | | 2015 | 3 | [42] |
| Rice | 200 | 0.118 | | 2016 | 8 | [46] |
| Rice | 1 | 0.232 | | 2018 | 7 | [51] |
| Rice | 1 | 0.154 | | 2018 | 7 | [51] |
| Rice | 1 | 0.063 | | 2018 | 7 | [51] |
| Rice | 7 | 0.058 | 0.012 | 2018 | 2 | [52] |
| Visceral meat | 5 | 0.158 | | 2010-2012 | 3 | [25] |
| Visceral meat | 110 | 0.054 | | 2010-2015 | 2 | [27] |
| Visceral meat | 69 | 0.0125 | | 2013-2014 | 6 | [36] |
| Visceral meat | 91 | 0.0147 | | 2013-2014 | 6 | [36] |
| Visceral meat | 51 | 0.009 | | 2013-2014 | 6 | [36] |
| Visceral meat | 33 | 0.007 | | 2013-2014 | 6 | [36] |
| **7. Dairy products** | | | | | | |
| Milk powder | 35 | 0.077 | | 2000 | 2 | [2] |
| Milk powder | 5 | 0.27 | 0.04 | 2000 | 2 | [3] |
| Milk powder | 10 | 0.01 | | 2006 | 2 | [9] |
| Milk powder | 210 | 0.013 | | 2006-2007 | 2 | [10] |
| Milk | 25 | 0.007 | | 2000 | 2 | [2] |
| Milk | 50 | 0.0075 | | 2005 | 2 | [7] |
| Milk | 210 | 0.004 | | 2006-2007 | 2 | [10] |
| Milk | 50 | 0.0075 | | 2007-2008 | 2 | [13] |
| Milk product | 1080 | 0.025 | | 2000 | 2 | [1] |
| Milk product | 1800 | 0.001 | 0.001 | 2009 | 1 | [21] |
| Milk product | 22 | 0.022 | | 2010-2015 | 2 | [27] |
| Yoghurt | 50 | 0.0075 | | 2007-2008 | 2 | [13] |
| **8. Eggs** | | | | | | |
| Duck egg | 50 | 0.0075 | | 2007-2008 | 2 | [13] |
| Duck egg | 50 | 0.0075 | | 2007-2008 | 2 | [13] |
| Eggs | 1080 | 0.027 | | 2000 | 2 | [1] |
| Eggs | 31 | 0.003 | | 2000 | 2 | [2] |
| Eggs | 50 | 0.0075 | | 2005 | 2 | [7] |
| Eggs | 10 | 0.01 | | 2006 | 2 | [9] |

| | | | | | | |
|---|---|---|---|---|---|---|
| White rice | 21 | 0.16 | | 2008 | 1 | [16] |
| White rice | 16 | 0.167 | 0.099 | 2008 | 1 | [17] |
| **2. Potatoes** | | | | | | |
| Potatoes | 1080 | 0.036 | | 2000 | 2 | [1] |
| Potatoes | 1800 | 0.009 | 0.004 | 2009 | 1 | [21] |
| **3. Legumes/Nuts** | | | | | | |
| Legumes | 1080 | 0.037 | | 2000 | 2 | [1] |
| Legumes | 20 | 0.053 | | 2000 | 2 | [2] |
| Legumes | 54 | 0.0094 | | 2005 | 2 | [7] |
| Legumes | 5 | 0.0018 | | 2009 | 2 | [18] |
| Legumes | 1800 | 0.011 | 0.004 | 2009 | 1 | [21] |
| peanut | 50 | 0.0075 | | 2005 | 2 | [7] |
| **4. Vegetables** | | | | | | |
| Moso bamboo shoot | 45 | 0.009 | | 2005 | 2 | [8] |
| Vegetables | 1080 | 0.039 | | 2000 | 2 | [1] |
| Vegetables | 53 | 0.014 | | 2000 | 2 | [2] |
| Vegetables | 5 | 0.028 | 0.014 | 2000 | 2 | [3] |
| Vegetables | 101 | 0.011 | | 2005 | 2 | [7] |
| Vegetables | 66 | 0.033 | | 2005 | 2 | [8] |
| Vegetables | 10 | 0.01 | | 2006 | 2 | [9] |
| Vegetables | 826 | 0.009 | | 2006-2007 | 2 | [10] |
| Vegetables | 1800 | 0.006 | 0.002 | 2009 | 1 | [21] |
| Vegetables | 87 | 0.02 | 0.056 | 2009-2010 | 2 | [22] |
| Vegetables | 75 | 0.024 | 0.093 | 2009-2010 | 2 | [22] |
| Vegetables | 79 | 0.015 | 0.02 | 2009-2010 | 2 | [22] |

| | | | | | | |
|---|---|---|---|---|---|---|
| Eggs | 601 | 0.007 | | 2006-2007 | 2 | [10] |
| Eggs | 50 | 0.0075 | | 2007-2008 | 2 | [13] |
| Eggs | 1800 | 0.007 | 0.002 | 2009 | 1 | [21] |
| Eggs | 522 | 0.02 | | 2010-2015 | 2 | [27] |
| Preserved egg | 50 | 0.0075 | | 2007-2008 | 2 | [13] |
| Preserved egg | 50 | 0.0075 | | 2005 | 2 | [7] |
| Quail egg | 50 | 0.0075 | | 2007-2008 | 2 | [13] |
| **9. Fish/Shrimp/Crab** | | | | | | |
| Aquatic Products | 1080 | 0.071 | | 2000 | 2 | [1] |
| Aquatic Products | 427 | 0.06 | | 2007-2008 | 2 | [14] |
| Aquatic Products | 1800 | 0.007 | 0.002 | 2009 | 1 | [21] |
| Aquatic Products | 106 | 0.005 | | 2015 | 1 | [43] |
| Crab | 50 | 0.097 | | 2005 | 2 | [7] |
| Crab | 50 | 0.097 | | 2007-2008 | 2 | [13] |
| Crab | 3 | 0.27 | 0.01 | 2011 | 5 | [30] |
| Crab | 8 | 0.103 | 0.067 | 2016 | 1 | [47] |
| Fish | 8 | 0.0075 | | 2002 | 1 | [5] |
| Fish | 16 | 0.077 | 0.031 | 2016 | 1 | [47] |
| Fish | 20 | 0.089 | 0.042 | 2016 | 1 | [47] |
| Fish products | 105 | 0.038 | 0.034 | 2016 | 1 | [44] |
| Fish products | 27 | 0.03 | 0.008 | 2016 | 1 | [44] |
| Freshwater fish | 33 | 0.009 | | 2000 | 2 | [2] |
| Freshwater fish | 5 | 0.02 | | 2000 | 2 | [3] |
| Freshwater fish | 50 | 0.009 | | 2005 | 2 | [7] |
| Freshwater fish | 10 | 0.01 | | 2006 | 2 | [9] |

| | | | | | | |
|---|---|---|---|---|---|---|
| Vegetables | 74 | 0.029 | | 2010-2015 | 2 | [27] |
| Vegetables | 112 | 0.024 | | 2010-2015 | 2 | [27] |
| Vegetables | 120 | 0.006 | | 2016 | 8 | [45] |
| **5. Fruits** | | | | | | |
| Fruits | 1080 | 0.018 | | 2000 | 2 | [1] |
| Fruits | 37 | 0.008 | | 2000 | 2 | [2] |
| Fruits | 5 | 0.008 | 0.002 | 2000 | 2 | [3] |
| Fruits | 10 | 0.01 | | 2006 | 2 | [9] |
| Fruits | 556 | 0.007 | | 2006-2007 | 2 | [10] |
| Fruits | 1800 | 0.008 | 0.001 | 2009 | 1 | [21] |
| Fruits | 255 | 0.021 | | 2010-2015 | 2 | [27] |
| Grape | 50 | 0.0075 | | 2005 | 2 | [7] |
| Peach | 50 | 0.0075 | | 2005 | 2 | [7] |
| Tangerine | 50 | 0.0075 | | 2005 | 2 | [7] |
| **6. Meats** | | | | | | |
| Beef | 50 | 0.0075 | | 2005 | 2 | [7] |
| Beef | 50 | 0.0075 | | 2007-2008 | 2 | [13] |
| Chicken | 50 | 0.0075 | | 2005 | 2 | [7] |
| Chicken | 31 | 0.023 | 0.015 | 2007 | 2 | [11] |
| Chicken | 50 | 0.0075 | | 2007-2008 | 2 | [13] |
| Chicken | 63 | 0.0078 | | 2013-2014 | 6 | [36] |
| Chicken | 47 | 0.005 | | 2013-2014 | 6 | [36] |
| Duck | 50 | 0.0075 | | 2005 | 2 | [7] |
| Duck | 30 | 0.02 | 0.023 | 2007 | 2 | [11] |
| Duck | 50 | 0.0075 | | 2007-2008 | 2 | [13] |
| Duck | 51 | 0.0075 | | 2005 | 2 | [7] |
| Freshwater fish | 50 | 0.009 | | 2007-2008 | 2 | [13] |
| Freshwater fish | 8 | 0.02 | | 2009-2011 | 2 | [23] |
| Freshwater fish | 16 | 0.03 | | 2014-2015 | 2 | [39] |
| Marine fish | 62 | 0.028 | | 2000 | 2 | [2] |
| Marine fish | 34 | 0.017 | | 2002 | 2 | [4] |
| Marine fish | 485 | 0.05 | | 2003-2005 | 2 | [6] |
| Marine fish | 10 | 0.028 | | 2006 | 2 | [9] |
| Marine fish | 50 | 0.0077 | | 2007-2008 | 2 | [13] |
| Marine fish | 5 | 0.0039 | | 2009 | 2 | [18] |
| Marine fish | 12 | 0.04 | | 2009-2011 | 2 | [23] |
| Marine fish | 22 | 0.026 | | 2010-2015 | 2 | [27] |
| Marine fish | 16 | 0.002 | 0.005 | 2013-2014 | 8 | [35] |
| Marine fish | 97 | 0.02 | | 2014-2015 | 2 | [39] |
| Marine fish | 15 | 0.505 | 0.365 | 2016 | 1 | [48] |
| Marine fish | 10 | 0.02 | | 2018 | 1 | [50] |
| Marine products | 68 | 0.171 | | 2000 | 2 | [2] |
| Marine products | 10 | 0.12 | 0.17 | 2000 | 2 | [3] |
| Shellfish | 9 | 0.216 | | 2002 | 2 | [4] |
| Shellfish | 43 | 0.063 | | 2003-2005 | 2 | [6 |
| Shellfish | 5 | 0.0977 | | 2009 | 2 | [18] |
| Shellfish | 27 | 0.055 | | 2010-2015 | 2 | [27] |
| Shellfish | 15 | 0.04 | | 2014-2015 | 2 | [39] |
| Shrimp | 50 | 0.011 | | 2005 | 2 | [7] |
| shrimp | 50 | 0.011 | | 2007-2008 | 2 | [13] |
| Shrimp | 10 | 0.09 | 0.035 | 2016 | 1 | [47] |
| Shrimp/Crab | 50 | 0.016 | | 2007-2008 | 2 | [13] |

| | | | | | | | | | | | |
|---|---|---|---|---|---|---|---|---|---|---|---|
| Livestock and poultry meat | 58 | 0.006 | 2000 | 2 | [2] | Shrimp/Crab | 8 | 0.15 | 2009-2011 | 2 | [23] |

[a], Methods of detection inorganic arsenic. 1 represents HPLC-ICP-MS; 2 represents Atomic Fluorescence Spectrometry; AFS; 3 represents HPLC-AFS; 4 represents HPLC-HG-AFS; 5 represents HPLC–UV-HG-AFS; 6 represents ICP-MS; 7 represents LC-ICP-MS; 8 represents LC-AFS. [b], study order means the chronological order of articles included in this study. [1]. (Li et al., 2006); [2]. (Yang et al., 2002); [3]. (Shi et al., 2000); [4]. (Li and Cang 2003); [5]. (Li et al., 2003); [6]. (Lin et al., 2007); [7]. (Zhou et al., 2008); [8]. (Zhao et al., 2006); [9]. (Lin 2007); [10]. (Zhang et al., 2008); [11]. (Xiao et al., 2008); [12]. (Meharg et al., 2008); [13]. (Zhou et al., 2009); [14]. (Liu et al., 2009); [15]. (Lu et al., 2010); [16]. (Meharg et al., 2009); [17]. (Zhu et al., 2008); [18]. (Wang 2011); [19]. (Cai et al., 2011); [20]. (Yun et al., 2010); [21]. (Feng 2016); [22]. (Chen et al., 2011); [23]. (Lin et al., 2012); [24]. (Liang et al., 2010); [25]. (Wang et al., 2013); [26]. (Wang et al., 2012); [27]. (Jiang et al., 2017); [28]. (Pan 2012); [29]. (Dai et al., 2014); [30]. (Zhang et al., 2013); [31]. (Shen 2013); [32]. (Huang et al., 2015); [33]. (Li et al., 2013); [34]. (Xie 2013); [35]. (Li et al., 2017b); [36]. (Hu et al., 2018); [37]. (Wang et al., 2016); [38]. (Xie 2014); [39]. (You et al., 2016); [40]. (Ma et al., 2017); [41]. (Ma et al., 2016); [42]. (Lin et al., 2015); [43]. (Fu et al., 2019); [44]. (Xue et al., 2017); [45]. (Jiao et al., 2017); [46]. (Tan et al., 2016); [47]. (Zhang et al., 2018); [48]. (Li et al., 2017a); [49]. (Chen et al., 2018); [50]. (Wang et al., 2018); [51]. (Su et al., 2018); [52]. (Liao et al., 2018). iAs, inorganic arsenic.

**Table S4. The number of studies (n) included in the present study in various periods.**

| Year | 2000–2004 | 2005–2009 | 2010–2014 | 2015–2019 | Total |
|---|---|---|---|---|---|
| *n* | 6 | 17 | 16 | 13 | 52 |

**Table S5 Consumption of different foods in different provinces in Chinese adults.**

| Consumption (g/day) | Cereals | | Legumes/ Nuts | Potatoes | Meat | Eggs | Dairy products | Aquatic products | Vegetables | Fruit |
|---|---|---|---|---|---|---|---|---|---|---|
| | Rice | Others (except rice) | | | | | | | | |
| **China** | 188.76 | 213.72 | 51.69 | 48.12 | 90.89 | 30.69 | 39.62 | 39.38 | 347.20 | 112.25 |
| **North** | | | | | | | | | | |
| Beijing | 99.71 | 321.88 | 132.23 | 48.17 | 115.28 | 62.26 | 77.93 | 16.19 | 491.70 | 237.74 |
| Hebei | 118.26 | 267.65 | 49.15 | 44.25 | 38.57 | 33.06 | 26.36 | 21.82 | 356.51 | 319.96 |
| Inner Mongolia | 55.25 | 377.67 | 73.07 | 202.01 | 116.43 | 55.62 | 108.16 | 4.61 | 245.87 | 107.60 |
| Shanxi* | 77.40 | 245.34 | 18.90 | 18.90 | 37.26 | 28.22 | 40.27 | 8.22 | 208.22 | 128.49 |
| Tianjin* | 99.71 | 223.30 | 13.01 | 13.01 | 69.04 | 46.03 | 46.85 | 45.48 | 315.34 | 199.18 |
| **Northeast** | | | | | | | | | | |
| Heilongjiang | 203.04 | 154.63 | 58.97 | 99.74 | 48.61 | 46.27 | 9.75 | 27.14 | 314.02 | 102.16 |
| Jilin | 288.72 | 404.35 | 100.32 | 144.20 | 114.57 | 55.64 | 17.54 | 27.95 | 492.66 | 74.67 |
| Liaoning | 295.79 | 183.91 | 254.61 | 63.94 | 93.18 | 50.54 | 52.67 | 16.19 | 259.26 | 197.50 |
| **East** | | | | | | | | | | |
| Anhui* | 198.85 | 155.67 | 17.26 | 17.26 | 62.19 | 28.77 | 29.32 | 29.59 | 255.89 | 104.66 |
| Fujian | 250.39 | 61.91 | 49.06 | 74.13 | 138.70 | 23.38 | 48.48 | 200.27 | 434.04 | 163.96 |
| Jiangsu | 16.30 | 347.74 | 102.48 | 47.59 | 135.39 | 37.88 | 72.63 | 84.35 | 385.23 | 172.09 |
| Jiangxi | 277.77 | 45.01 | 33.33 | 31.40 | 87.97 | 18.27 | 48.56 | 22.40 | 249.83 | 12.21 |
| Shandong* | 118.26 | 205.58 | 12.60 | 12.60 | 55.62 | 43.01 | 49.86 | 30.69 | 243.56 | 166.30 |
| Shanghai | 218.51 | 85.20 | 75.42 | 20.11 | 165.31 | 40.54 | 100.30 | 110.42 | 499.58 | 141.00 |
| Zhejiang | 377.61 | 98.84 | 63.33 | 29.09 | 99.90 | 43.83 | 70.83 | 168.65 | 449.94 | 207.50 |
| **Central** | | | | | | | | | | |
| Henan | 28.70 | 502.87 | 31.43 | 53.57 | 31.97 | 36.46 | 2.88 | 6.26 | 322.92 | 61.44 |
| Hubei | 293.87 | 140.45 | 61.54 | 57.77 | 70.33 | 33.99 | 3.28 | 60.60 | 521.44 | 33.64 |

| | | | | | | | | | | |
|---|---|---|---|---|---|---|---|---|---|---|
| Hunan | 261.78 | 139.13 | 44.92 | 25.43 | 135.31 | 28.21 | 9.62 | 48.87 | 603.81 | 3.39 |
| **South** | | | | | | | | | | |
| Guangdong | 125.03 | 177.71 | 23.28 | 17.85 | 123.10 | 18.80 | 36.46 | 67.99 | 244.93 | 38.69 |
| Guangxi | 295.18 | 65.70 | 37.28 | 15.60 | 147.14 | 22.55 | 4.31 | 37.71 | 500.18 | 3.96 |
| Hainan* | 210.11 | 54.55 | 5.89 | 5.89 | 79.18 | 13.15 | 11.78 | 73.43 | 241.92 | 75.89 |
| **Southwest** | | | | | | | | | | |
| Chongqing* | 240.19 | 127.76 | 21.23 | 21.23 | 107.67 | 27.67 | 40.00 | 27.12 | 364.11 | 108.49 |
| Guizhou* | 265.72 | 76.20 | 15.07 | 15.07 | 89.04 | 12.06 | 14.52 | 6.03 | 249.04 | 76.16 |
| Sichuan | 240.19 | 74.43 | 79.52 | 87.65 | 121.20 | 20.42 | 11.61 | 16.47 | 548.97 | 103.45 |
| Tibet* | 148.56 | 590.08 | 10.00 | 10.00 | 107.12 | 8.49 | 58.63 | 1.37 | 67.67 | 16.71 |
| Yunnan* | 268.00 | 73.92 | 13.29 | 13.29 | 81.10 | 13.70 | 15.07 | 10.14 | 269.86 | 73.43 |
| **Northwest** | | | | | | | | | | |
| Gansu* | 214.82 | 192.30 | 15.48 | 15.48 | 49.32 | 20.82 | 36.71 | 5.48 | 202.19 | 134.25 |
| Ningxia | 214.82 | 200.32 | 95.22 | 104.30 | 91.44 | 19.01 | 19.70 | 23.18 | 359.83 | 175.05 |
| Qinghai | 56.92 | 391.58 | 6.23 | 92.68 | 82.48 | 13.85 | 62.09 | 5.07 | 452.80 | 58.90 |
| Shaanxi | 77.40 | 365.93 | 83.32 | 84.63 | 59.74 | 30.12 | 48.56 | 8.23 | 328.41 | 21.75 |
| Xinjiang* | 214.82 | 273.67 | 4.93 | 4.93 | 63.56 | 18.63 | 53.43 | 8.77 | 283.56 | 159.45 |

* The rice consumption data is equal to the average value of multiple neighboring provinces.

**Table S6. The number of CHD deaths and prevalent cases attributed to food-borne inorganic arsenic (iAs) intake in different regions in China in 2016.**

| Provinces | Deaths, in thousands (95% UI) | Age-standardized mortality rate, per 100 000 (95% UI ) | Prevalent cases, in thousands (95% UI ) | Age-standardized prevalence rate, per 100 000 (95% UI ) |
|---|---|---|---|---|
| China | 177.52(172.06-183.19) | 15.4(14.93-15.89) | 1668.09(1582.95-1750.61) | 144.72(137.33-151.88) |
| **North** | | | | |
| Beijing | 3.45(3-3.87) | 17.8(15.49-19.99) | 43.48(41.25-45.78) | 224.51(213.02-236.39) |
| Hebei | 13.07(11.59-14.52) | 21.46(19.02-23.83) | 109.38(103.97-115.02) | 179.57(170.69-188.83) |
| Inner Mongolia | 4.46(3.97-5.02) | 20.31(18.06-22.87) | 36.88(34.99-38.87) | 167.84(159.21-176.9) |
| Shanxi | 0.83(0.74-0.92) | 2.67(2.36-2.95) | 12.27(11.61-12.92) | 39.37(37.26-41.47) |
| Tianjin | 1.62(1.42-1.81) | 11.62(10.17-13.03) | 14.9(14.17-15.64) | 107.06(101.79-112.4) |
| **Northeast** | | | | |
| Heilongjiang | 9.96(8.73-11.06) | 29.14(25.56-32.37) | 69.51(65.85-73) | 203.41(192.72-213.64) |
| Jilin | 12.06(10.9-13.24) | 50.51(45.67-55.44) | 75.81(71.32-80.54) | 317.57(298.76-337.36) |
| Liaoning | 16.34(14.34-18.11) | 41.75(36.66-46.29) | 136.52(129.64-143.69) | 348.94(331.36-367.28) |
| **East** | | | | |
| Anhui | 4.53(4.13-4.95) | 8.86(8.09-9.69) | 43.76(41.44-46.28) | 85.65(81.11-90.57) |
| Fujian | 4.32(3.85-4.81) | 13.63(12.16-15.2) | 61.52(58.52-64.68) | 194.35(184.87-204.33) |
| Jiangsu | 2.08(1.88-2.31) | 3.01(2.72-3.35) | 39.66(37.64-41.78) | 57.48(54.55-60.55) |
| Jiangxi | 4.06(3.63-4.51) | 11.21(10.03-12.46) | 39.93(37.72-42.14) | 110.38(104.26-116.5) |
| Shandong | 10.03(8.96-11.07) | 12.14(10.84-13.4) | 85.67(80.83-90.46) | 103.69(97.84-109.48) |
| Shanghai | 2.68(2.37-3) | 12.28(10.83-13.71) | 44.11(41.67-46.42) | 201.8(190.62-212.35) |
| Zhejiang | 6.82(6.06-7.72) | 14.02(12.46-15.87) | 117.78(109.76-125.23) | 242.04(225.55-257.34) |
| **Central** | | | | |
| Henan | 12.73(11.59-13.86) | 16.86(15.35-18.36) | 100.77(95.51-105.87) | 133.46(126.5-140.22) |
| Hubei | 10(9.09-10.97) | 20.11(18.27-22.05) | 98.94(93.36-104.44) | 198.91(187.7-209.98) |
| Hunan | 15.45(13.95-17.61) | 27.69(25-31.55) | 121.39(114.5-128.29) | 217.53(205.19-229.9) |
| **South** | | | | |
| Guangdong | 1.54(1.39-1.68) | 1.68(1.52-1.84) | 27.23(25.92-28.61) | 29.74(28.3-31.25) |
| Guangxi | 8.45(7.56-9.42) | 22.2(19.88-24.75) | 68.98(65.27-72.59) | 181.29(171.54-190.78) |
| Hainan | 0.58(0.51-0.64) | 7.83(6.98-8.73) | 5.85(5.54-6.17) | 79.45(75.23-83.8) |
| **Southwest** | | | | |
| Chongqing | 3.29(2.94-3.67) | 12.77(11.42-14.23) | 37.04(34.92-39.23) | 143.73(135.49-152.22) |
| Guizhou | 3.11(2.69-3.53) | 11.26(9.75-12.79) | 30.65(28.96-32.53) | 110.94(104.82-117.71) |
| Sichuan | 11.4(10.11-12.77) | 16.41(14.56-18.39) | 127.53(119.97-135.28) | 183.67(172.79-194.84) |
| Tibet | 0.16(0.14-0.19) | 6.31(5.44-7.53) | 1.25(1.18-1.33) | 49.85(47.02-52.82) |
| Yunnan | 4.19(3.76-4.71) | 10.91(9.81-12.26) | 36.72(34.58-38.87) | 95.65(90.08-101.24) |
| **Northwest** | | | | |
| Gansu | 3.11(2.78-3.47) | 14.33(12.82-16.01) | 29.11(27.37-30.81) | 134.3(126.3-142.18) |
| Ningxia | 1.23(1.09-1.38) | 22.51(19.9-25.26) | 11.39(10.78-11.99) | 208.66(197.5-219.74) |
| Qinghai | 0.66(0.58-0.74) | 13.91(12.19-15.62) | 5.04(4.73-5.35) | 105.78(99.26-112.38) |
| Shaanxi | 5.16(4.59-5.82) | 15.9(14.13-17.91) | 37.6(34.97-40.16) | 115.79(107.68-123.68) |

| | | | | |
|---|---|---|---|---|
| Xinjiang | 4.66(4.15-5.22) | 25.09(22.35-28.09) | 29.82(28.24-31.45) | 160.51(151.97-169.29) |

**Table S7. The annual count(AC) and DALY per case of lung, bladder, and skin cancer.**

| Provinces | LCAC | LC DALY per case | SCAC | SC DALY per case | BCAC | BC DALY per case |
|---|---|---|---|---|---|---|
| **China** | 17165.62 | 18.31(17.35-19.02) | 14993.08 | 11.16(9.55-12.01) | 1391.47 | 7.11(6.69-8.21) |
| **North** | | | | | | |
| Beijing | 280.05 | 11.05(9.03-13.06) | 244.61 | 3.33(2.17-4.8) | 22.7 | 3.62(2.68-4.5) |
| Hebei | 917.45 | 20.61(17.07-24.38) | 801.33 | 34.17(23.87-43.89) | 74.37 | 9.21(7.17-12.49) |
| Inner Mongolia | 310.65 | 18.09(15.24-21.39) | 271.34 | 26.19(19.57-32.12) | 25.18 | 7.84(6.47-9.41) |
| Shanxi | 320.98 | 19.35(15.4-23.55) | 280.36 | 21.35(16.17-26.57) | 26.02 | 6.41(4.98-8.6) |
| Tianjin | 153.06 | 15.06(12.72-25.21) | 133.68 | 4.65(3.58-6.57) | 12.41 | 5.29(4.14-6.5) |
| **Northeast** | | | | | | |
| Heilongjiang | 491.32 | 29.22(24.29-34.66) | 429.13 | 12.01(9.66-15.79) | 39.83 | 8.06(6.6-9.73) |
| Jilin | 581.47 | 18.62(15.09-22) | 507.87 | 50.81(33.78-65.71) | 47.13 | 7.4(5.77-8.87) |
| Liaoning | 762.9 | 20.24(16.82-24.08) | 666.34 | 11(8.12-13.4) | 61.84 | 7.15(5.3-8.62) |
| **East** | | | | | | |
| Anhui | 658.67 | 20.34(17.1-24.29) | 575.31 | 9.84(7.78-11.66) | 53.39 | 9.18(7.61-11.23) |
| Fujian | 662.3 | 19.73(16.27-23.86) | 578.47 | 8.7(6.86-11.3) | 53.69 | 7.31(5.77-9.72) |
| Jiangsu | 739.26 | 19.95(16.61-23.7) | 645.69 | 6.96(5.26-8.6) | 59.93 | 7.88(6.35-9.5) |
| Jiangxi | 603.1 | 20.86(17.77-24.55) | 526.77 | 14.81(12.01-17.66) | 48.89 | 9.23(7.66-11.68) |
| Shandong | 981.34 | 20.41(17.05-24.18) | 857.14 | 12.86(10.33-15.91) | 79.55 | 7.7(6.42-9.38) |
| Shanghai | 328.27 | 8.58(7.05-10.41) | 286.72 | 2.86(1.81-3.71) | 26.61 | 3.36(2.36-4.27) |
| Zhejiang | 1117.47 | 14.15(11.63-16.98) | 976.04 | 4.23(3.12-5.52) | 90.58 | 5.94(4.75-7.2) |
| **Central** | | | | | | |
| Henan | 1109.35 | 19.73(16.49-23.28) | 968.94 | 17.72(14.44-21.44) | 89.93 | 9.65(7.71-14.68) |
| Hubei | 934.86 | 21.48(17.96-25.48) | 816.54 | 9.78(7.99-12.31) | 75.78 | 8(6.54-9.64) |
| Hunan | 1083.87 | 22.93(19.22-27.72) | 946.69 | 25.83(19.54-31.67) | 87.86 | 11.44(9.04-15.88) |
| **South** | | | | | | |
| Guangdong | 1036.34 | 16.91(14.12-20.12) | 905.18 | 4.8(3.63-6.2) | 84.01 | 5.89(4.86-7.4) |
| Guangxi | 779.46 | 18.98(15.95-22.78) | 680.81 | 10.27(8.42-12.72) | 63.18 | 8.09(6.3-12.92) |
| Hainan | 105.06 | 15.67(12.6-19.65) | 91.77 | 7.68(5.71-11.21) | 8.52 | 6.76(5.14-9.29) |
| **Southwest** | | | | | | |
| Chongqing | 408.15 | 17.48(13.99-21.81) | 356.5 | 7.99(6.25-10.73) | 33.09 | 7.08(5.56-9.2) |
| Guizhou | 468.64 | 13.44(11.05-16.07) | 409.33 | 23.3(17.32-29.38) | 37.99 | 8.2(6.62-10.11) |
| Sichuan | 1216.73 | 25.59(20.71-30.05) | 1062.73 | 20.4(16.45-24.46) | 98.63 | 11.62(9.2-14.18) |
| Tibet | 41.38 | 12.98(10.7-15.68) | 36.15 | 23.61(18.39-29.81) | 3.35 | 10.98(8.36-20.02) |
| Yunnan | 649.55 | 15.47(13.04-18.5) | 567.34 | 13.15(10.11-16.08) | 52.65 | 7.27(6.04-9.06) |
| **Northwest** | | | | | | |
| Gansu | 349.56 | 14.38(11.99-17.18) | 305.32 | 7.43(6.16-8.71) | 28.34 | 5.76(4.75-7.05) |
| Ningxia | 105.82 | 16.01(12.94-19.45) | 92.43 | 3.68(3.03-4.46) | 8.58 | 4.86(3.83-6.07) |
| Qinghai | 77.65 | 15.99(12.97-19.05) | 67.82 | 10.62(8.69-12.69) | 6.29 | 7.83(6.13-11.02) |
| Shaanxi | 488.78 | 15.8(12.62-19.64) | 426.92 | 15.78(12.89-19.07) | 39.62 | 7.4(5.8-10.11) |
| Xinjiang | 360.74 | 10.71(8.83-12.66) | 315.09 | 4.13(3.3-4.95) | 29.24 | 4.27(3.23-6.51) |

**Reference**

Cai, J.,Wang, B.,Li, X., 2011. Investigation on the health status of corn and rice in Jilin in 2009. Chinese Journal of Disease Control. 15:1068-1070

Chen, B.,Guan, S.,Gauan, H.,Huang, X.,Ke, Z.,Tang, K., et al., 2011. Status quo of heavy metal pollution in native grown vegetables in Nanhai District of Foshan city and risk analysis on human health. South China Journal of Preventive Medicine. 37:3-8

Chen, H.,Tang, Z.,Wang, P.,Zhao, F.J., 2018. Geographical variations of cadmium and arsenic concentrations and arsenic speciation in Chinese rice. Environ Pollut. 238:482-490. http://dx.doi.org/10.1016/j.envpol.2018.03.048.

Dai, S.,Yang, H.,Mao, X.,Qiu, J.,Liu, Q.,Wang, F., et al., 2014. Evaluation of arsenate content of rice and rice bran purchased from local markets in the People's Republic of China. J Food Prot. 77:665-669. http://dx.doi.org/10.4315/0362-028x.Jfp-13-344.

Feng, H. Study on pollution level and exposure assessment of inorganic arsenic in Chinese diet. Wuhan Polytechnic University; 2016

Fu, L.,Lu, X.,Niu, K.,Tan, J.,Chen, J., 2019. Bioaccumulation and human health implications of essential and toxic metals in freshwater products of Northeast China. Sci Total Environ. 673:768-776. http://dx.doi.org/10.1016/j.scitotenv.2019.04.099.

Hu, Y.,Zhang, W.,Chen, G.,Cheng, H.,Tao, S., 2018. Public health risk of trace metals in fresh chicken meat products on the food markets of a major production region in southern China. Environ Pollut. 234:667-676. http://dx.doi.org/10.1016/j.envpol.2017.12.006.

Huang, Y.,Wang, M.,Mao, X.,Qian, Y.,Chen, T.,Zhang, Y., 2015. Concentrations of Inorganic Arsenic in Milled Rice from China and Associated Dietary Exposure Assessment. J Agric Food Chem. 63:10838-10845. http://dx.doi.org/10.1021/acs.jafc.5b04164.

Jiang, Y.,Meng, H.,Chen, H.,Cheng, H.,Tang, Z., 2017. Risk assessment of arsenic contamination in main foods and dietary exposure of residents in Guangxi. Chinese Journal of Food Hygiene. 29:745-749. http://dx.doi.org/10.13590/j.cjfh.2017.06.022.

Jiao, H.,Liu, Y.,Liu, S.,Liu, Z.,Cao, X.,Yang, X., et al., 2017. Investigation and exposure Assessment of Different forms of Arsenic in dietary vegetables in Jinan in 2016. Modern Preventive Medicine. 44:2154-2156+2178

Li, F.,Cang, G., 2003. Physical and chemical index analysis of food contaminant monitoring in Jiangsu Province in 2002. Chinese Journal of Health Laboratory Technology.635-636. http://dx.doi.org/10.3969/j.issn.1004-8685.2003.05.049.

Li, G.,Zheng, M.,Zhu, Y., 2013. Study on arsenic levels and health risks in rice in Fujian Province. Asian Journal of Ecotoxicology. 8:148-155. http://dx.doi.org/10.7524/AJE.1673-5897.20130103001.

Li, J.,Sun, C.,Zheng, L.,Jiang, F.,Wang, S.,Zhuang, Z., et al., 2017a. Determination of trace metals and analysis of arsenic species in tropical marine fishes from Spratly islands. Mar Pollut Bull. 122:464-469. http://dx.doi.org/10.1016/j.marpolbul.2017.06.017.

Li, M.,Pu, Y.,Wang, Z.,LI, S.,Li, J.,Liu, J., et al., 2017b. Monitoring results of 4 arsenic forms in 5 types of seafood sold in Beijing. Occupation and Health.

33:2655-2659. http://dx.doi.org/10.13329/j.cnki.zyyjk.2017.0794.
Li, W.,Wei, C.,Zhang, C.,van Hulle, M.,Cornelis, R.,Zhang, X., 2003. A survey of arsenic species in Chinese seafood. Food Chem Toxicol. 41:1103-1110. http://dx.doi.org/10.1016/s0278-6915(03)00063-2.
Li, X.W.,Gao, J.Q.,Wang, Y.F.,Chen, J.S., 2006. [2000 Chinese total dietary study--the dietary arsenic intakes]. Wei Sheng Yan Jiu. 35:63-66. http://dx.doi.org/10.3969/j.issn.1000-8020.2006.01.018.
Liang, F.,Li, Y.,Zhang, G.,Tan, M.,Lin, J.,Liu, W., et al., 2010. Total and speciated arsenic levels in rice from China. Food Addit Contam Part A Chem Anal Control Expo Risk Assess. 27:810-816. http://dx.doi.org/10.1080/19440041003636661.
Liao, N.,Seto, E.,Eskenazi, B.,Wang, M.,Li, Y.,Hua, J., 2018. A Comprehensive Review of Arsenic Exposure and Risk from Rice and a Risk Assessment among a Cohort of Adolescents in Kunming, China. Int J Environ Res Public Health. 15. http://dx.doi.org/10.3390/ijerph15102191.
Lin, D.,Wu, L.,Mai, J.,Zhang, Q.,Yang, F., 2012. Analysis of aquatic Product pollution in Panyu District, Guangzhou from 2009 to 2011. Journal of Tropical Medicine. 12:1150-1153
Lin, J., 2007. Investigation of inorganic arsenic contamination in some foods in Ruian city in 2006. Shanghai Journal of Preventive Medicine.351. http://dx.doi.org/10.3969/j.issn.1004-9231.2007.07.016.
Lin, K.,Lu, S.,Wang, J.,Yang, Y., 2015. The arsenic contamination of rice in Guangdong Province, the most economically dynamic provinces of China: arsenic speciation and its potential health risk. Environ Geochem Health. 37:353-361. http://dx.doi.org/10.1007/s10653-014-9652-1.
Lin, Y.,Yan, Z.,Wang, Y.,Zhao, Q.,Zhou, Y.,Xiao, C., et al., 2007. Risk and early warning measures of heavy metal pollution of import and export aquatic products in a city. Modern Preventive Medicine.813-814. http://dx.doi.org/10.3969/j.issn.1003-8507.2007.05.005.
Liu, F.,Dai, J.,Qiu, F., 2009. Investigation on heavy metal pollution of Aquatic products in Shenzhen. Practical Preventive Medicine. 16:1487-1488. http://dx.doi.org/10.3969/j.issn.1006-3110.2009.05.061.
Lu, Y.,Dong, F.,Deacon, C.,Chen, H.J.,Raab, A.,Meharg, A.A., 2010. Arsenic accumulation and phosphorus status in two rice (Oryza sativa L.) cultivars surveyed from fields in South China. Environ Pollut. 158:1536-1541. http://dx.doi.org/10.1016/j.envpol.2009.12.022.
Ma, L.,Wang, L.,Jia, Y.,Yang, Z., 2016. Arsenic speciation in locally grown rice grains from Hunan Province, China: Spatial distribution and potential health risk. Sci Total Environ. 557-558:438-444. http://dx.doi.org/10.1016/j.scitotenv.2016.03.051.
Ma, L.,Wang, L.,Tang, J.,Yang, Z., 2017. Arsenic speciation and heavy metal distribution in polished rice grown in Guangdong Province, Southern China. Food Chem. 233:110-116. http://dx.doi.org/10.1016/j.foodchem.2017.04.097.
Meharg, A.A.,Lombi, E.,Williams, P.N.,Scheckel, K.G.,Feldmann, J.,Raab, A., et al., 2008. Speciation and localization of arsenic in white and brown rice grains. Environ Sci Technol. 42:1051-1057. http://dx.doi.org/10.1021/es702212p.
Meharg, A.A.,Williams, P.N.,Adomako, E.,Lawgali, Y.Y.,Deacon, C.,Villada, A., et al., 2009. Geographical variation in total and inorganic arsenic content of polished (white) rice. Environ Sci Technol. 43:1612-1617. http://dx.doi.org/10.1021/es802612a.

Pan, H. Study on inorganic Arsenic exposure in Rice in China. Wuhan University of Technology; 2012

Shen, T., 2013. Current situation and potential health risk assessment of heavy metal pollution of green food rice in Tianmen City. Hubei Plant Protection.53-57

Shi, H.,Hu, J.,Ding, G., 2000. Study on the limit standard of inorganic Arsenic in food. Anhui Journal of Preventive Medicine.433

Su, Z.,Hu, S.,Cai, W.,Yang, X.,Wang, J.,Fan, J., et al., 2018. To establish the detection method of inorganic arsenic and its application in the detection of rice matrix. Chinese Journal of Preventive Medicine. 52:994-1002. http://dx.doi.org/10.3760/cma.j.issn.0253-9624.2018.10.006.

Tan, H.,Zhou, H.,Li, J., 2016. Monitoring and analysis of arsenic in rice in Jiangxi Province. Chinese Journal of Health Laboratory Technology. 26:722-723+726

Wang, H.,Yi, L.,Wang, H.,Liu, B.,Zhi, Y.,Zhao, H., 2018. Contamination of lead, cadmium, mercury and inorganic arsenic in Marine products in qinhuangdao waters and risk assessment. Modern Preventive Medicine. 45:4443-4446

Wang, J.,Zhang, R.,Song, C.,Zhang, X., 2012. Monitoring and result analysis of heavy metals in livestock meat in Hohhot from 2010 to 2012. Chinese Journal of Health Laboratory Technology. 22:2977-2978

Wang, M.,Lian, Y.,Pang, X.,Xi, J.,Wu, Y., 2016. Analysis and risk Assessment of heavy metal Pollution in late rice in Ningbo city. China Rice. 22:65-68. http://dx.doi.org/10.3969/j.issn.1006-8082.2016.04.017.

Wang, Y., 2011. Analysis on the contamination of lead and arsenic in food samples in Funing County. Modern Preventive Medicine. 38:1426-1427+1429

Wang, Z.,Zhang, X.,Li, D.,Hu, M.,Chang, H.,Zhao, C., et al., 2013. Analysis of monitoring results of heavy metals and harmful elements in food in Dazhou from 2010 to 2012. Chinese Journal of Health Laboratory Technology. 23:3404-3409

Wei, J.,Gao, J.,Cen, K., 2019. Levels of eight heavy metals and health risk assessment considering food consumption by China's residents based on the 5th China total diet study. Sci Total Environ. 689:1141-1148. http://dx.doi.org/10.1016/j.scitotenv.2019.06.502.

Xiao, Q.,Deng, K.,Liu, F.,Mo, H., 2008. Monitoring of heavy metal contamination of raw poultry and livestock food in Shenzhen in 2007. Practical Preventive Medicine. 15:1760-1763. http://dx.doi.org/10.3969/j.issn.1006-3110.2008.06.030.

Xie, K. Detection and exposure assessment of total arsenic and arsenic morphologies in rice from major grain producing areas in China. Wuhan Polytechnic University; 2013

Xie, W. Current status of heavy metal enrichment in 50 rice species. Central South University; 2014

Xue, M.,Gong, L.,Wang, Z.,Jin, Q.,Ren, R.,Wu, H., 2017. Investigation and Analysis on pollution status of 7 toxic elements in ready-to-eat fish products sold in Hangzhou in 2016. Chinese Journal of Health Laboratory Technology. 27:1948-1951+1955

Yang, H.,Liang, C.,Dong, S.,Cang, G.,Bian, J.,Jiang, L., et al., 2002. Hygienic standards for monitoring and limiting inorganic arsenic in food in some areas of China. Journal of Hygiene Research. 31:431-434. http://dx.doi.org/10.3969/j.issn.1000-8020.2002.06.012.

You, Z.,Yang, Y.,Huang, X.,Zhang, J.,Gao, Y.,Xu, Q., 2016. Monitoring and Analysis of heavy metals in raw aquatic products of catering Industry in Guizhou

Province in 2014-2015. Journal of Qiannan Ethnic Medical College. 29:204-207
Yun, H.,Zhang, L.,Li, X.,Zhao, Y.,Wu, Y., 2010. Study on determination of inorganic Arsenic in rice. Journal of Hygiene Research. 39:316-320. http://dx.doi.org/10.19813/j.cnki.weishengyanjiu.2010.03.015.
Zhang, L.,Peng, S.,Qi, L.,Tian, M.,Chen, R.,Zhao, Y., et al., 2008. Analysis of monitoring results of food contaminants in Shanghai from 2006 to 2007. Journal of Environmental and Occupational Medicine.337-341. http://dx.doi.org/10.3969/j.issn.1006-3617.2008.04.003.
Zhang, W.,Guo, Z.,Song, D.,Du, S.,Zhang, L., 2018. Arsenic speciation in wild marine organisms and a health risk assessment in a subtropical bay of China. Sci Total Environ. 626:621-629. http://dx.doi.org/10.1016/j.scitotenv.2018.01.108.
Zhang, W.,Wang, W.X.,Zhang, L., 2013. Arsenic speciation and spatial and interspecies differences of metal concentrations in mollusks and crustaceans from a South China estuary. Ecotoxicology. 22:671-682. http://dx.doi.org/10.1007/s10646-013-1059-8.
Zhao, R.,Zhao, M.,Wang, H.,Taneike, Y.,Zhang, X., 2006. Arsenic speciation in moso bamboo shoot--a terrestrial plant that contains organoarsenic species. Sci Total Environ. 371:293-303. http://dx.doi.org/10.1016/j.scitotenv.2006.03.019.
Zhou, X.,Chen, Z.,Wang, L.,Wang, R.,Pan, G.,Yu, X., 2008. Status and analysis of contamination of lead, cadmium, arsenic and aluminum in food in Shaoxing, Zhejiang province in 2005. Disease Surveillance.100-106. http://dx.doi.org/10.3784/j.issn.1003-9961.2008.02.014.
Zhou, X.,Zhang, W.,Chen, Z.,Wang, J.,Wang, L.,Wang, R., 2009. Study on heavy metal Pollution level of Animal-derived food in Shaoxing. Chinese Journal of Health Laboratory Technology. 19:898-902
Zhu, Y.G.,Sun, G.X.,Lei, M.,Teng, M.,Liu, Y.X.,Chen, N.C., et al., 2008. High percentage inorganic arsenic content of mining impacted and nonimpacted Chinese rice. Environ Sci Technol. 42:5008-5013. http://dx.doi.org/10.1021/es8001103.
